# Biomonitoring 2.0 Refined: observing local change through metaphylogeography using a community-based eDNA metabarcoding monitoring network

**DOI:** 10.1186/s12915-025-02284-x

**Published:** 2025-07-01

**Authors:** Andrew C. Riley, Michael Wright, Teresita M. Porter, V. Carley Maitland, Donald J. Baird, Mehrdad Hajibabaei

**Affiliations:** 1 Centre for Biodiversity Genomics & Department of Integrative Biology, University of Guelph, Guelph, ON Canada; 2 University of New Brunswick, Fredericton, New Brunswick, Canada

**Keywords:** Citizen science, Phylogeography, Populations, Haplotypes, Conservation

## Abstract

**Background:**

Biological data at different levels of organization is essential to support actions to mitigate the current biodiversity crisis. DNA metabarcoding is an established method to detect species/genus level taxa from bulk samples leading the way for a Biomonitoring 2.0 framework. Biomonitoring 2.0 Refined adds another dimension to Biomonitoring 2.0—high-throughput, scalable DNA metabarcoding with a higher resolution at the intraspecific level. Intraspecific diversity is key to understanding the distribution and movement of local populations for conservation efforts. Gaining reliable intraspecific information from metabarcoding data, however, is challenging due to qualitative/quantitative issues that can impact validity of the inference.

**Results:**

Samples collected for the STREAM community-based monitoring project were used to perform an intraspecific genetic variation analysis on benthic arthropods. We targeted two non-overlapping cytochrome c oxidase subunit 1 mitochondrial DNA amplicons to assess the reproducibility of our results. Samples from the Rocky Mountains were grouped into four regions separated by mountain ranges. Significant separation (PERMANOVA, *p* value < 0.05) of Sørensen dissimilarity between regions was observed for community and intraspecific levels, fitting the expectation that mountains are barriers to dispersal. Two of the regions showed significant spatial structuring (Mantel test, *p* value < 0.05) at the intraspecific level, while all regions showed significant structuring at the community level. Results were consistent across both amplicons.

**Conclusions:**

We show that DNA metabarcoding is applicable to intraspecific diversity analysis and it is robust to different amplicons. This paves the way for Biomonitoring 2.0 Refined, which can provide much needed fine-scale biodiversity data for ecological assessments and conservation.

**Supplementary Information:**

The online version contains supplementary material available at 10.1186/s12915-025-02284-x.

## Background

We are witnessing an unprecedented pressure on earth’s ecosystems and their species due to climate change and various other anthropogenic activities, such as land use change and resource extraction [1, 2]. The global biodiversity crisis and species decline are major global issues demanding action at local, national, and global scales [3, 4]. Consequently, regulations and policies have been developed that require activities with impacts on nature and biodiversity be reported [5, 6]. To support these policies, robust and scalable tools are needed to detect and quantify biodiversity.

A spectrum of scientific approaches and tools are available for understanding biodiversity and ecological change [7–11]. These tools focus on different levels of organization and/or target different taxonomic groups. For example, biomonitoring approaches may focus on bioindicators known to be sensitive to environmental perturbations and can act as an early warning system for ecological change [12]. In the aquatic environment, species such as fish, diatoms, and macroinvertebrates have been used as bioindicators [13–16]. Researchers, regulatory agencies, and ecological/environmental assessment companies use different sampling techniques and data analysis approaches for biomonitoring projects. While many iterations of these approaches exist, most of them focus on generating a taxonomic matrix of species/taxa occurring at a site and then comparing them to reference sites (pristine sites) to derive a diagnosis for the sites under investigation [17]. These programs require spatiotemporal analyses to obtain statistically meaningful results and to generate potential predictive models.

In practice, however, conventional biomonitoring approaches suffer from critical shortcomings [9, 18]. The main issue is difficulty in identification of species from environmental samples [19]. For example, identification of larval specimens of macroinvertebrates based on their morphology requires taxonomic expertise and is labor-intensive. In some cases, making a species-level identification is impossible and many taxa are routinely identified to the genus or family level; identifications may even vary from one technician to the next [20]. Because of these limitations, conventional biomonitoring programs lack the resolution, scalability, reproducibility, and rapid turnaround time that is needed to make decisions in a timely manner.

DNA-based approaches such as DNA barcoding and environmental DNA (eDNA) metabarcoding have been successfully utilized to aid biomonitoring. The term Biomonitoring 2.0 was introduced to describe the use of high-throughput sequencing to generate metabarcodes from environmental samples for scalable biomonitoring [21]. Biomonitoring 2.0 helped democratize access to biodiversity information at scale, super-charging the development applications for aquatic, terrestrial, and even aerial monitoring [22–28]. It has played a role in conservation and remediation, being adopted by researchers in the academic, government, and private sector, sparking calls for the development of new tools, databases, and standards [29–31]. Typically, similar sequences are grouped into operational taxonomic units (OTUs), a species proxy, and the overall community diversity is investigated. However, this approach still aims to provide taxonomic data at the species/genus level similar to what is generated when using conventional morphological methods.

Leveraging intraspecific diversity from DNA metabarcoding data allows us to quantify biodiversity with a much finer level of resolution [32–34]. Environmental stressors directly impact individual organisms, but through interaction networks, these effects can impact the structure of the local population and the broader community [35]. Observing changes in intraspecific diversity may be more sensitive for monitoring environmental stressors than community diversity [36]. Furthermore, intraspecific diversity can be used to identify barriers to dispersal, as distinct population structuring suggests that the mixing of populations is not occurring or being limited [37, 38]. As a result, conservation efforts have been focused on phylogeography, where phylogenetic patterns are associated with geographic features, to better understand the local adaptation of a species or lack thereof [39, 40]. Typically, phylogeographic analyses are limited to a single species. However, by using DNA metabarcoding data, the phylogeographic patterns of multiple species can be simultaneously analyzed. This type of analysis has been named metaphylogeography [41]. Metaphylogeography has been used to study intraspecific diversity between habitats [42], marine barriers [38, 41], and anthropogenic stress on rivers [43].

In terrestrial biogeographical differentiation, mountains have been identified as the third most important factor after climate and continental drift, because they act primarily as a barrier to dispersal [44]. However, mountains are considered to be semi-permeable barriers, because depending on their dispersal capabilities, some species may move freely cross mountains [45]. How frequently species disperse across a barrier will determine if species cohesion is maintained [42]. Previous studies have found genetic structuring in species within and separated by mountains [46–50]. This makes mountains an excellent landscape to study barriers that are acting at both the community and intraspecific level.

However, a finer-scale analysis of diversity comes with greater potential to be influenced by sequencing errors [41, 51, 52]. Intraspecific diversity analyses will be restricted to comparisons of highly similar sequences in smaller groups, and differentiating true variability and sequencing error becomes more challenging as sequence similarity increases [53]. If errors are included in an analysis, they could lead to over estimations of haplotypes as well as alter patterns in genetic variation. Therefore, there is a need to validate intraspecific diversity derived from DNA metabarcoding data. Previous studies have used mock communities of aquatic macroinvertebrates to validate the recovery of true haplotypes from metabarcoding data [52, 54]. Elbrecht et al. specifically focused on developing a filtering strategy to recover haplotypes [52], while Serrana and Watanabe focused on laboratory processing [54]. Both studies were able to recover the majority of haplotypes present; however, neither study was able to recover all expected haplotypes without unexpected ones. Studies have also validated intraspecific diversity from DNA metabarcoding using field samples by comparing results to previous population studies of selected species [37, 41]. Similarly, both studies recovered previously identified haplotypes as well as haplotypes unique to the DNA metabarcoding data.

Mock communities and comparisons to previous studies have established the ability of metabarcoding data to retrieve intraspecific diversity level information. However, it is difficult to determine if mock communities will be representative of the true complexity of real communities, and comparisons to existing studies require previous studies on the same species identified in the DNA metabarcoding data and in the same geographic area. Alternative methods for validating intraspecific diversity from metabarcoding data are necessary to further establish its utility.

Intraspecific diversity has the potential to add another layer of information to DNA metabarcoding data. We use the term Biomonitoring 2.0 Refined to highlight the importance of including individual species analysis and the context it can provide for community analyses, and vice versa. In this study, samples of benthic macroinvertebrates in an eDNA metabarcoding framework from the STREAM project, a citizen science initiative, were used to generate species-level data to compare community β-diversity and intraspecific genetic variation patterns. We selected samples collected in the eastern Rocky Mountains to validate patterns against geographical information associated with being major barriers to dispersal. We also demonstrate the robustness of using multiple primer sets in validating intraspecific-level data. This work sets the stage for combining the taxonomically oriented ecological assessments with species and population oriented conservation biology, hence providing a significant advancement in our ability to monitor biodiversity at the time it is needed most critically.

## Results

### Metabarcode data processing and clustering

**Fig. 1:**
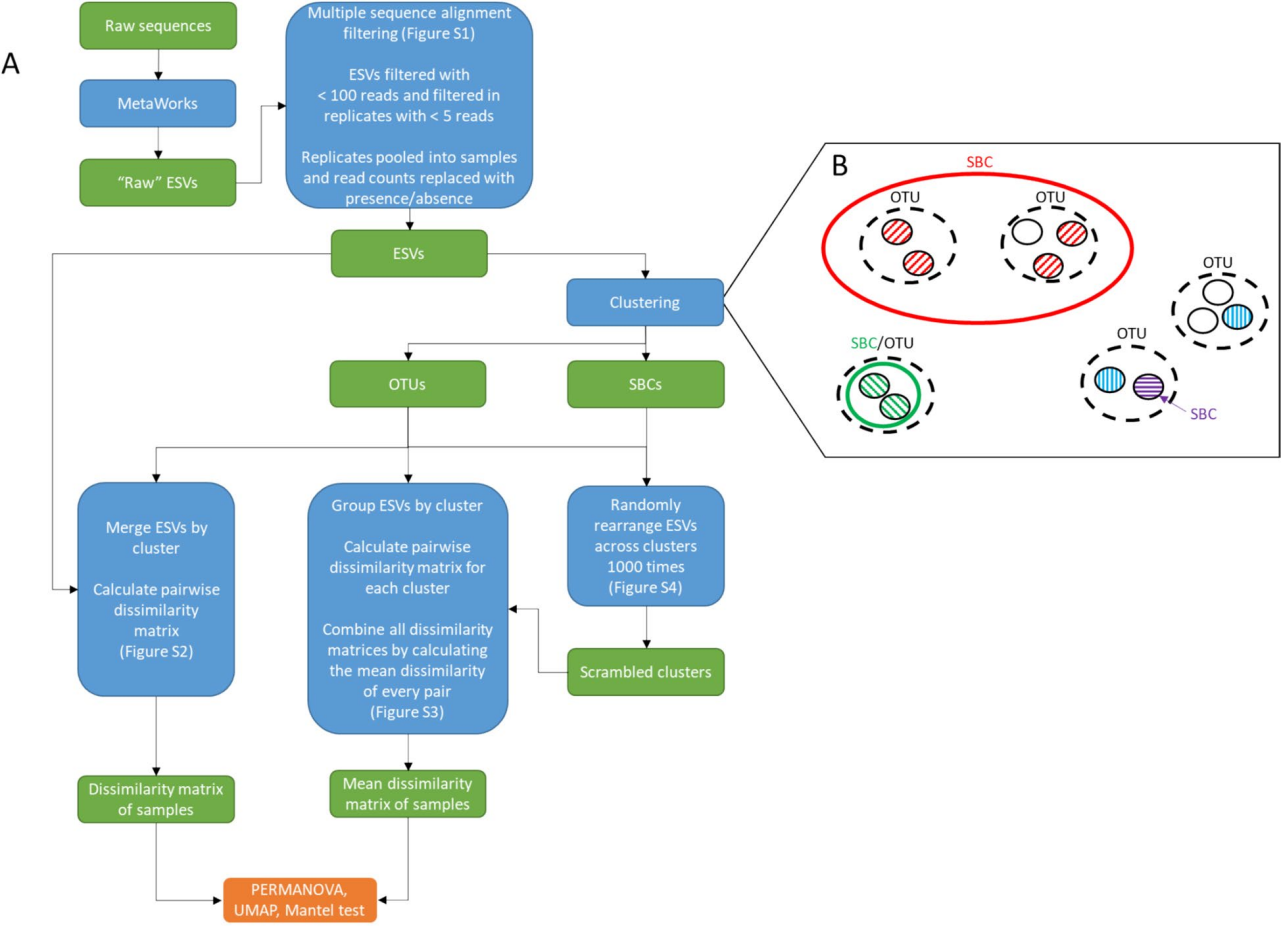
Processing steps to go from raw sequences to dissimilarity matrices for community and intraspecific diversity comparisons. Flow diagram where green boxes are data or data structures, blue boxes are data processing steps, and the orange box represents final statistical and visualization steps (**A**). Sequence space of clustered ESVs (**B**). Each circle represents an ESV, striped circles represent ESVs assigned to a species, while white circles represent ESVs without a species assignment. Black dashed ellipses represent ESVs clustered into OTUs, which are defined by a sequence similarity threshold. Colored solid ellipses represent ESVs clustered into SBCs, which are defined as clusters that include all and only ESVs assigned to one species, the sequence similarity threshold can vary between these clusters. Any species with only one assigned ESV will be an SBC. Abbreviations: ESV, exact sequence variant; OTU, operational taxonomic unit; SBC, species bound cluster (Additional file 2: Figs. S1–S4)

A flow diagram of data processing steps is shown in Fig. 1A. In total, 131,701,837 raw reads were acquired across 138 samples. After filtering and clustering steps were performed in the MetaWorks pipeline [55], 8136 F230R exact sequence variants (ESVs), also referred to as amplicon sequence variants [32] or zero-radius OTUs [56], and 22,035 MLJG ESVs were obtained for Arthropoda. After multiple sequence alignment (MSA) filtering for length and frameshifts was performed, 6818 F230R ESVs remained that were 229 nt in length and 14,496 MLJG ESVs remained that were 313 nt in length. Finally, after filtering ESVs with fewer than 100 reads and removing ESVs with fewer than 5 reads in each sample replicate, 2026 (14,579,800 reads) F230R ESVs and 1567 (5,940,237 reads) MLJG ESVs remained.

ESVs were treated as haplotype proxies and clustered into species proxies using two different methods (Fig. 1B). The first method clustered ESVs into OTUs using a fixed minimal similarity threshold; there were 964 and 833 OTUs for F230R and MLJG, respectively. The second method clustered ESVs into species bound clusters (SBCs) using a variable minimal similarity threshold that identified clusters that contained all and only ESVs assigned to one species. F230R had 857 ESVs with species-level bootstrap support (sBP) of 0.8 or greater, assigned to 425 unique species, while MLJG had 603 ESVs assigned to 240 unique species. Of these species, 416 (892 ESVs) and 238 (633 ESVs) were recovered as SBCs for F230R and MLJG, respectively. ESVs without a species assignment can be clustered into SBCs. The majority of SBCs were in the orders Diptera, Ephemeroptera, Plecoptera, and Trichoptera (Additional file 1: Table S1).

### Region groups

**Fig. 2:**
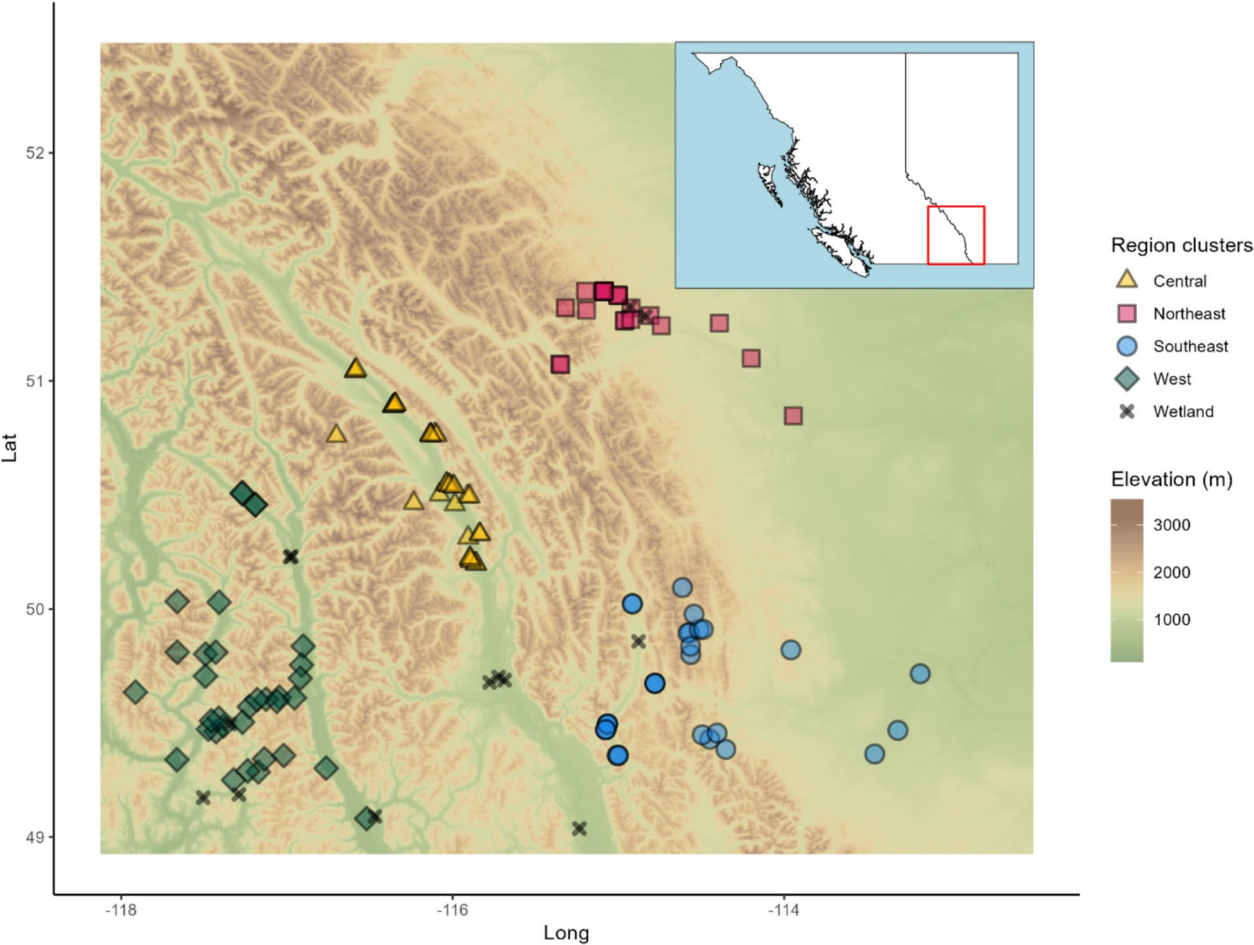
Sample collection locations on elevation map of Rocky Mountains between British Columbia and Alberta. Sample collection locations are split into four region groups identified using k-means clustering. Wetland sample locations are marked with an X

The 138 samples collected in the Rocky Mountains were grouped into regions using k-means clustering with four cluster centers; this was the only number of centers that resulted in stable clusters (Additional file 2: Fig. S5). Additionally, the total within-cluster sum of squares plateaus around four cluster centers (Additional file 2: Fig. S6). The four region groups were named central, northeast, southeast, and west based on their location (Fig. 2). Thirteen samples were specifically designated as wetlands; these samples were omitted from further analysis. Their inclusion/exclusion did not change the region groups. For F230R and MLJG respectively, there were 327 and 151 ESVs, 193 and 83 OTUs, and 100 and 38 SBCs that were unique to these samples. There were 36 samples in the central group (35 samples for MLJG), 25 samples in the northeast group, 30 samples in the southeast group, and 34 samples in the west group. Based on visual inspection of Fig. 2, there is a clear separation of the west, central, and northeast region groups by mountain ranges. The southeast region group is within and on the same side as the most eastern range of mountains as the northeast region group.

### Region group community β-diversity

To compare community β-diversity between region groups, the Sørensen dissimilarity between pairs of samples was calculated for ESVs (haplotype-level community), ESVs merged into OTUs (species-level community), and ESVs merged into SBCs (species-level community). PERMANOVA analysis found that diversity was significantly different between region groups for both the F230R and MLJG COI amplicons for all community representations (Additional file 1: Table S2). The pseudo *F*-statistics, the variation between groups divided by the variation within groups, were 10.4, 9.6, and 8.9 for F230R and 8.6, 8.0, and 8.5 for MLJG for ESVs, OTUs, and SBCs, respectively. The statistical dispersion of the dissimilarities within region groups was never found to be significantly different for either amplicon or community representation (Additional file 1: Table S3), suggesting that significant difference between region groups is not due to differences in variation distribution. Pairwise PERMANOVAs were also performed between region groups, and in all cases every region pair was found to be significantly different (Additional file 1: Table S4). Comparisons of the northeast and southeast regions always had the lowest pseudo *F*-statistic, while every comparison of the northeast and west regions had the highest pseudo *F*-statistic, with the exception of F230R for SBCs where it was the second highest.

### Region group intraspecific genetic variation

To compare intraspecific genetic variation between region groups, ESVs were grouped by clusters and the Sørensen dissimilarity between pairs of samples was calculated to create dissimilarity matrices for each cluster. Sørensen dissimilarity was selected to use a consistent metric between intraspecific genetic variation and community β-diversity. A mean dissimilarity matrix was calculated from all of the dissimilarity matrices. This ensures that measured dissimilarities comes from comparisons of ESVs within the same cluster and are therefore representative of intraspecific genetic variation. When referencing OTUs and SBCs that have been processed this way, they will be referred to as intraspecific OTUs and intraspecific SBCs. Of the OTUs, 165 (755 ESVs) and 115 (529 ESVs) met the criteria (3 or more samples in at least 2 region groups) for intraspecific genetic variation analysis for F230R and MLJG, respectively. For the SBCs, 79 (383 ESVs) F230R and 53 (284 ESVs) MLJG met the criteria for intraspecific genetic variation analysis. Between the two species proxies, 54 F230R clusters and 38 MLJG clusters were identical. After calculating the mean dissimilarity matrices for each species proxy and removing samples with missing pairwise values, 121 samples remained for F230R (central = 36, northeast = 25, southeast = 27, west = 33) and 101 samples remained for MLJG (central = 30, northeast = 18, southeast = 22, west = 31).

Intraspecific OTUs and SBCs were compared to ESV community β-diversity reduced to the same samples; this will be referred to as community ESVs. ESVs were selected because they had the strongest β-diversity separation between region groups, based on pseudo *F*-statistics, of all community comparisons for both F230R and MLJG. Again, PERMANOVA analysis was found to be significantly different in all cases (Additional file 1: Table S5), while the dispersion was found to be not significantly different in all cases (Additional file 1: Table S6). Pseudo *F*-statistics for F230R were 25.8 and 24.6 for the intraspecific SBCs and OTUs, while the pseudo *F*-statistic for the community ESVs was lower at 10.7. Similarly, for MLJG, the pseudo *F*-statistics were 13.3 and 19.7 for the intraspecific SBCs and OTUs, compared to 9.3 for the community ESVs. Pairwise PERMANOVAs were also performed on all dissimilarity matrices and comparisons between all regions were significantly different (Additional file 1: Table S7). In all cases, the lowest pseudo *F*-statistics were observed between the northeast and southeast clusters, while in all cases the highest pseudo *F*-statistics were observed between the northeast and west clusters, with the exception of F230R for intraspecific SBCs where it was the second highest.

**Fig. 3:**
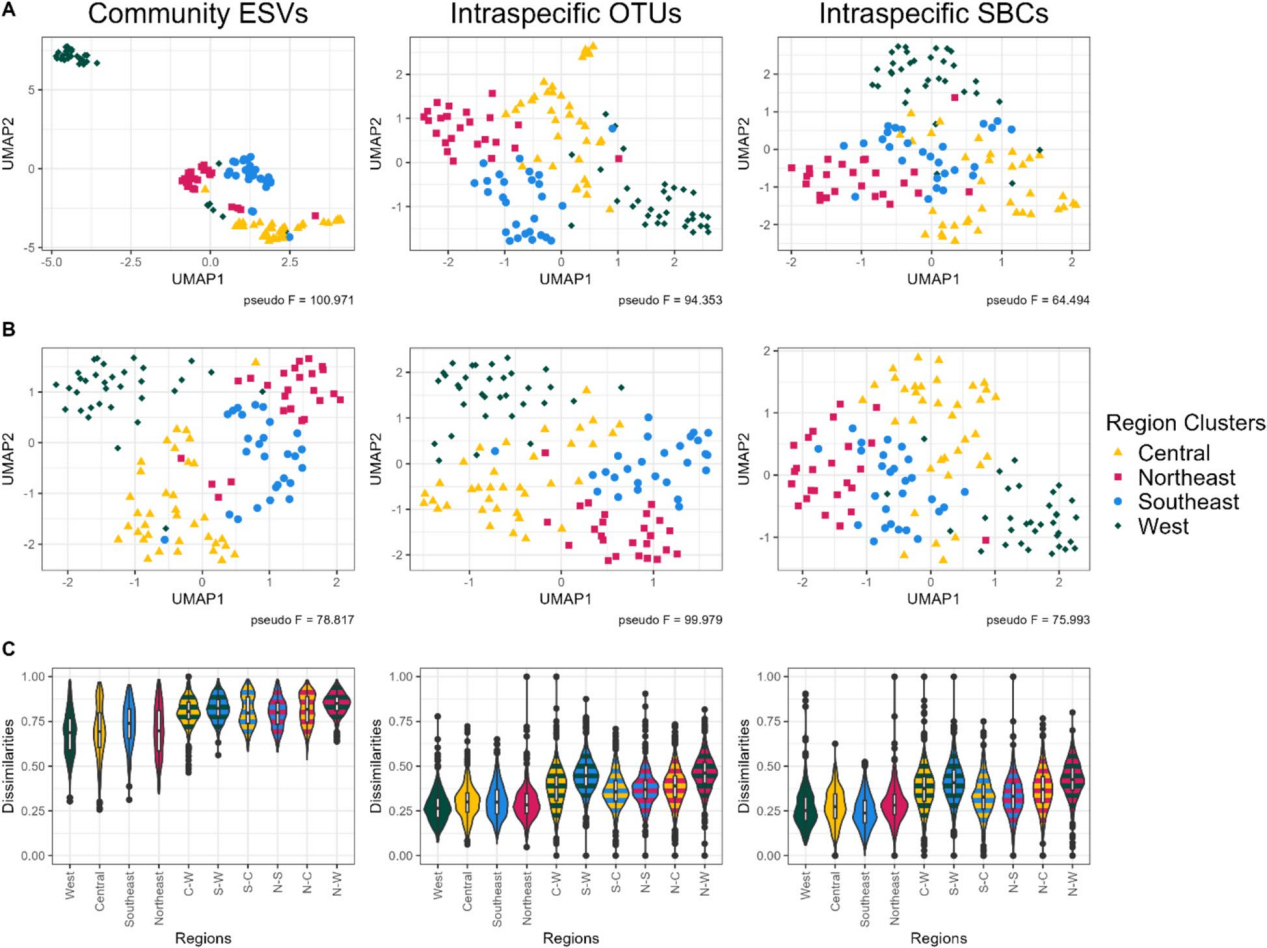
Intraspecific genetic variation separates region groups with dissimilarity patterns that differ from community β-diversity. Uniform manifold approximation and projection (UMAP) using 15 neighbors (**A**), using max neighbors (number of samples − 1) (**B**), and violin plots of dissimilarities (**C**) were generated to compare the community ESVs to intraspecific OTUs and SBCs from amplicon F230R. Pseudo *F*-statistics were calculated using PERMANOVA for the separation of region groups based on UMAP ordinations. All PERMANOVA tests were significant (*p* value < 0.05). Abbreviations: C-W, central-west; ESV, exact sequence variant; N–C, northeast-central; N-S, northeast-southeast; N-W, northeast-west; OTU, operational taxonomic unit; SBC, species bound cluster; S-C, southeast-central; S-W, southeast-west

To visualize intraspecific genetic variation and community β-diversity, ordination of dissimilarity matrices was performed using uniform manifold approximation and projection (UMAP) [57]. UMAP can be described as creating a weighted k-neighbor graph, then a lower dimensional layout of this graph is computed. The number of neighbors used effects whether local structure (low neighbors) or global structure (high neighbors) is emphasized [58]. UMAP was run using the default number of neighbors (15), as well as the maximum number of neighbors (*n* − 1, *n* = number of samples). UMAP plots of F230R matrices can be found in Fig. 3A and B. Additionally, violin plots were created to compare the dissimilarities within and between region groups (Fig. 3C). PERMANOVA was used to evaluate how well the ordinations separated the region groups using Euclidean distance. Separation of region groups is apparent for both intraspecific SBCs and OTUs. However, at 15 neighbors, the community ESVs has much tighter clusters. When the maximum number of neighbors is used, the spread of the region groups appears more consistent and the pseudo *F*-statistics increases for both intraspecific SBCs and OTUs. Additionally, the dissimilarities within region groups appear to be shifted towards the maximum dissimilarity (1.0) for the community ESVs when compared to the intraspecific SBCs and OTUs. Similar results were observed for MLJG (Additional file 2: Fig. S7); however, separation of region groups from intraspecific genetic variation was not as strong, especially in the case of the intraspecific SBCs. Correlation between intraspecific OTUs, intraspecific SBCs, and community ESVs ranged from 0.30 to 0.74 for F230R and 0.27 to 0.63 for MLJG (Additional file 1: Table S8). The correlation between amplicons was 0.27 and 0.41 for intraspecific SBCs and OTUs, respectively. All correlations were significant.

**Fig. 4:**
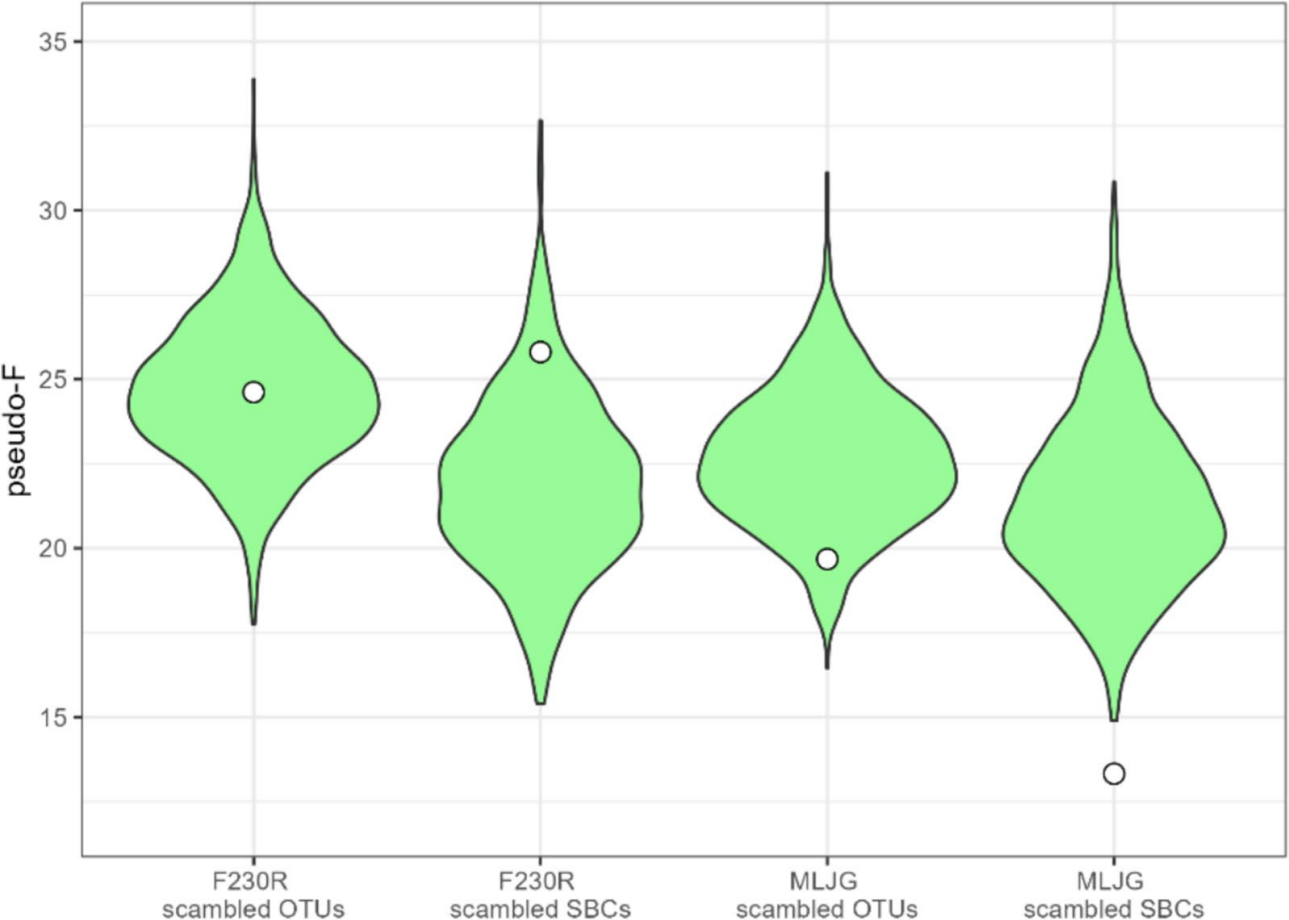
Region group separation of scrambled OTUs and SBCs is more consistent with intraspecific genetic variation. Region group separation was measured using the pseudo *F*-statistics from PERMANOVA analysis and violin plots were used to compare the distribution of the scrambled species pseudo *F*-statistics. All pseudo *F*-statistics were significant (*p* value < 0.05). White circles mark the equivalent intraspecific OTUs and SBCs pseudo *F*-statistic. Abbreviations: OTU, operational taxonomic unit; SBC, species bound cluster

The method for creating intraspecific mean dissimilarity matrices differed from how the community dissimilarity matrices were generated. To verify if the higher pseudo *F*-statistics observed for the intraspecific genetic variation was due to better separation or data manipulation, ESVs in species proxies were randomly rearranged to create scrambled clusters (Additional file 2: Fig. S4). These scrambled clusters are intended to not represent any true species, and any observed patterns should reflect the community level comparisons rather than the intraspecific level. This was done 1000 times using both OTUs and SBCs for each amplicon. These scrambled clusters were used to create mean dissimilarity matrices mimicking intraspecific OTUs and SBCs; these will be referred to as scrambled OTUs and SBCs. Distributions of the pseudo *F*-statistic from the scrambled OTUs and SBCs were compared to the pseudo *F*-statistics of the intraspecific OTUs and SBCs (Fig. 4). Both F230R intraspecific OTUs and SBCs and the MLJG intraspecific OTUs fell within the distribution of pseudo *F*-statistics of the scrambled OTUs and SBCs, while the MLJG intraspecific SBCs fell below. This suggests that the improvement in separation may be due to how the mean dissimilarity matrices were generated, rather than a true improvement in region separation for the intraspecific genetic variation over the community β-diversity.

### Spatial structure of β-diversity and genetic variation within region groups

**Fig. 5:**
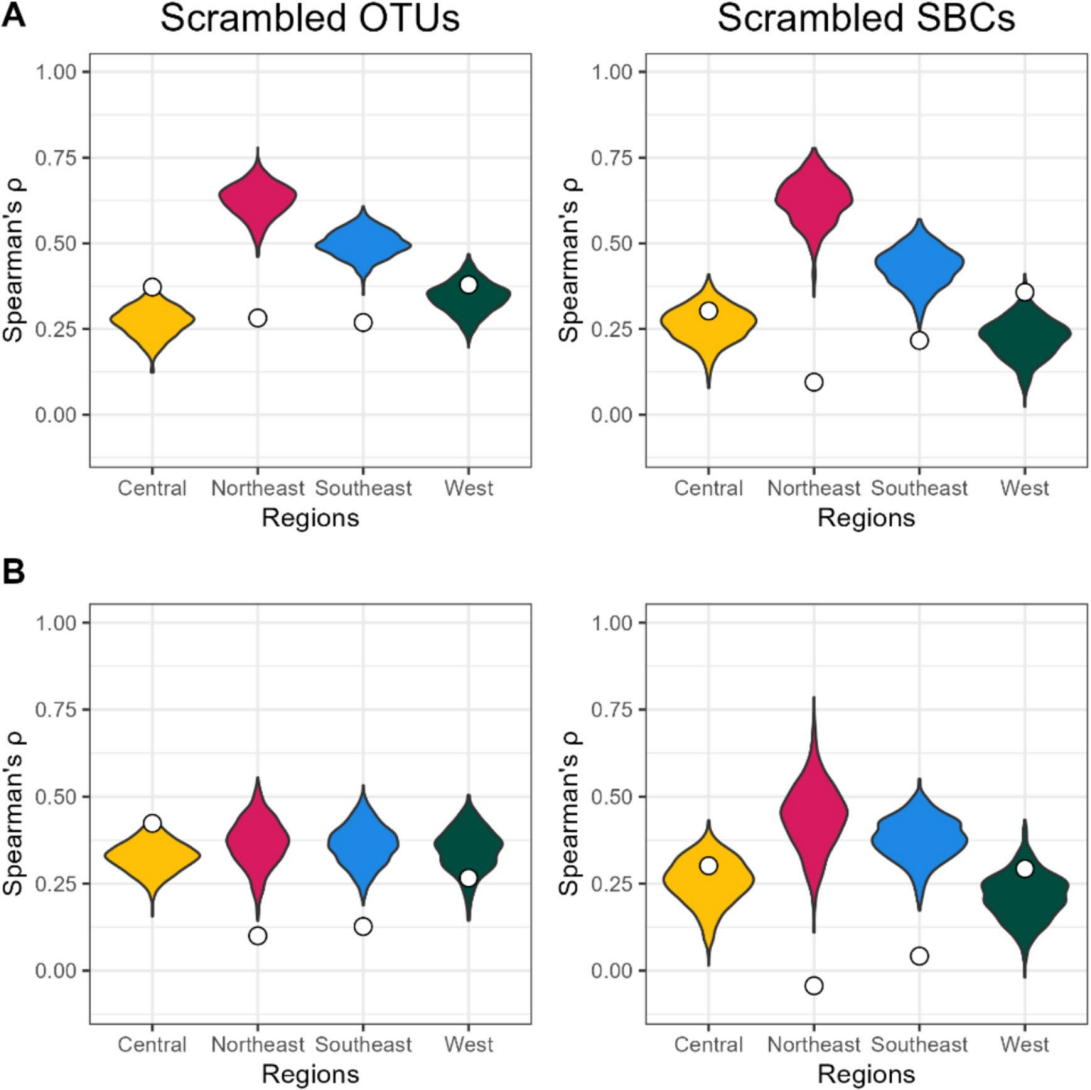
Spatial structuring within region groups varies between scrambled OTUs and SBCs and intraspecific genetic variation. Spatial structuring was measured using the Spearman’s rank correlation between scrambled OTUs and SBCs and geographic distance matrices within region groups. Violin plots were used to compare distribution of scrambled OTUs and SBCs correlations for both F230R (**A**) and MLJG (**B**). White circles indicate the equivalent intraspecific OTUs and SBCs correlation. Abbreviations: OTU, operational taxonomic unit; SBC, species bound cluster

Spatial structuring was determined by calculating Spearman’s rank correlation between the geodesic distances of sample locations and the dissimilarity matrices within each region (Fig. 5). Intraspecific OTUs and SBCs correlations and significance can be found in Additional file 1: Table S9 and a summary of scrambled OTUs and SBCs correlations can be found in Additional file 1: Table S10. For the intraspecific OTUs and SBCs, the northeast and southeast regions always had the lowest correlation, and they were the only regions with correlations not significantly different from zero. This suggests that there is limited or no spatial structuring for intraspecific genetic variation in these regions. Conversely, the scrambled OTUs and SBCs generally had the highest correlations in the northeast and southeast regions, and all of the observed correlations were higher than the intraspecific OTUs and SBCs correlations. Not every correlation was significantly different from zero. In the case of the central and west regions, all of the intraspecific OTUs and SBCs correlations fell into the correlation ranges of the scrambled OTUs and SBCs.

### Individual intraspecific genetic variation within region groups

To determine if there was significant region group separation within individual species, PERMANOVA analysis was run on the dissimilarity matrix of every individual OTU and SBC. From Table 1, the majority of species had significant region group separation for F230R, while MLJG had just under the majority.

**Table 1:** Number of individual species proxies with significant region group separation

Amplicons	OTUs	SBCs
F230R	99 (60%)	49 (62%)
MLJG	46 (40%)	25 (47%)

Significant region group separation was determined by a *p* value < 0.05 from a PERMANOVA analysis on the dissimilarity matrix of each individual species. *p* values were adjusted with the false discovery rate method

*Abbreviations:* *OTU* Operational taxonomic unit, *SBC* Species bound cluster

**Fig. 6:**
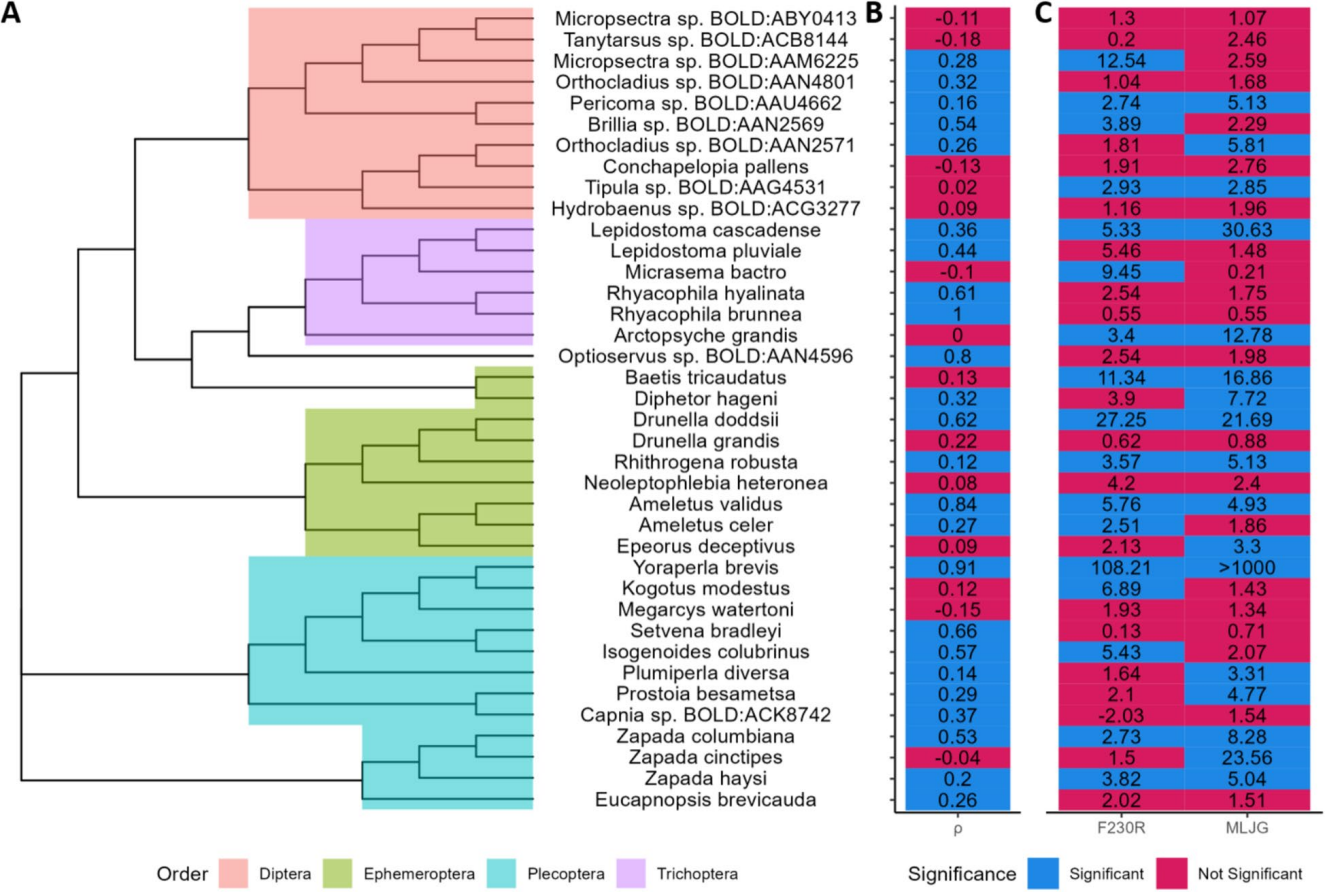
Comparison of F230R and MLJG ESVs assigned to the same SBC. Neighbor-joining tree was created using the most abundant MLJG ESV for each species (**A**). *Optioservus* sp. BOLD:ANN4596 was the only species belonging to the order Coleoptera. Correlations between genetic variation of F230R and MLJG for each species, which was calculated using Spearman’s rank correlation and significance was measured using the Mantel test (**B**). A significant Mantel test suggests that the correlation between dissimilarity matrices is not zero. Pseudo *F*-statistics of the region separation for F230R and MLJG based on a PERMANOVA analysis (**C**). A significant PERMANOVA indicates that centroids and/or distribution of genetic variation is different between region groups. Significance was determined using *p* value adjusted with the false discovery rate method (*p* value < 0.05). Abbreviations: ESV, exact sequence variant; SBC, species bound cluster

Since the SBCs were assigned taxonomically, comparisons could be made between amplicons of the SBCs with the same assignment. Of the 79 F230R and 53 MLJG SBCs, 38 species were found in both amplicons. Spearman’s correlation between the dissimilarity matrices of each amplicon was calculated and the PERMANOVA results were compared to see if SBCs had consistent results between amplicons (Additional file 1: Table S11). Of the 38 SBCs, 24 had significant correlations, 26 had amplicons that were in agreement if there was significant or not significant region group separation, and 16 fell into both groups (Fig. 6). All 7 species with a correlation over 0.6 were in agreement on region group separation significance.

**Fig. 7:**
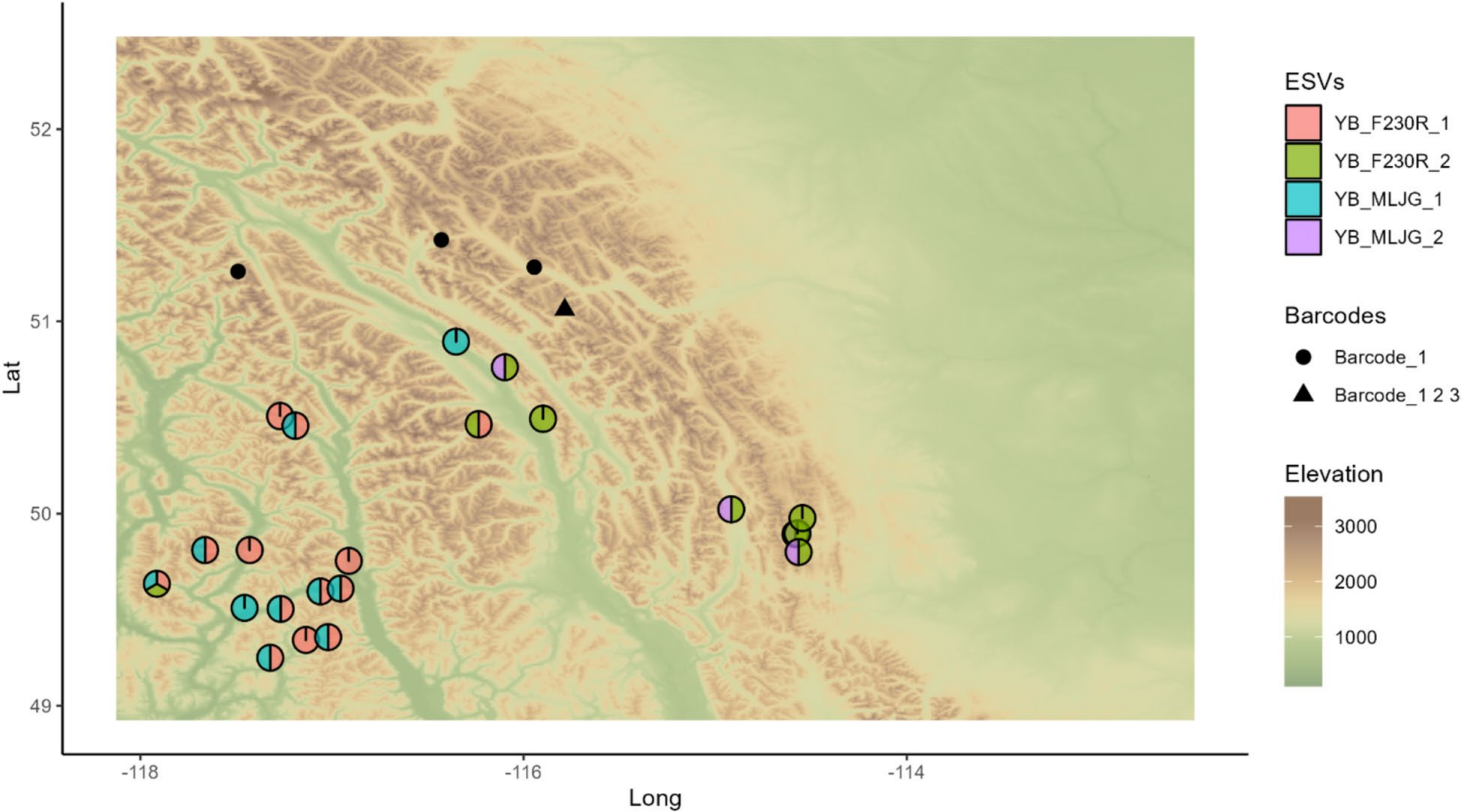
Collection locations of *Yoraperla brevis* ESVs and barcodes. Pie charts represent the presence of ESVs at each sample location. Abbreviations: ESV, exact sequence variant

*Yoraperla brevis* was selected for further analysis because its F230R and MLJG amplicons had the strongest separation between region groups, based on pseudo *F*-statistics, and the second highest correlation between amplicons (Fig. 6). Additionally, Hughes et al. performed a genetic structure study on *Y. brevis* in montane streams [59], see Discussion for details. Both F230R and MLJG amplicons had two unique ESVs with one nucleotide difference; these will be referred as YB_F230R_1, YB_F230R_2, YB_MLJG_1, and YB_MLJG_2. YB_F230R_1 and YB_MLJG_1 were predominantly found in the west region cluster, while YB_F230R_2 and YB_MLJG_2 were predominantly found in the southeast region group; all ESVs were found in the central region group (Fig. 7). In BOLD systems taxonomy database, 8 specimens were identified as *Y. brevis* belonging to one BIN (retrieved December 12, 2024) [60]. From these specimens, there were three unique barcodes, ignoring gaps; these will be referred to as Barcode_1, Barcode_2, and Barcode_3 (Additional file 2: Fig. S8). Barcode_1, Barcode_2, and Barcode_3 were all collected in the same location; specimens collected at other locations were all Barcode_1 (Fig. 7). One specimen fell outside the study area. YB_F230R_1 and YB_MLJG_1 are both identical to Barcode_1, YB_F230R_2 and YB_MLJG_2 are both identical to Barcode_2, and YB_MLJG_1 is identical to Barcode_3 (Additional file 2: Fig. S8). Although data is limited, there is clear consistency between ESVs, barcodes, and their geographic location.

## Discussion

The goal of Biomonitoring 2.0 Refined is to gain a deeper understanding of the available information content in DNA metabarcoding data. In this study, we show that DNA metabarcoding data could be used to identify patterns in community β-diversity and intraspecific genetic variation across mountains. Patterns at the community and intraspecific level were similar and consistently observed for both amplicons that were analyzed. To our knowledge, this is the first study to use multiple non-overlapping amplicons to validate intraspecific genetic variation in broadly targeted DNA metabarcoding data.

### Mountain biogeography and phylogeography

Investigating patterns at the community and intraspecific level simultaneously allows for the investigations of DNA metabarcoding data at different timescales [61–63]. Patterns at the community level will emerge over longer periods of time when compared to intraspecific-level patterns. In our Rocky Mountain sites, there is consistent separation of region groups using community β-diversity and intraspecific genetic variation. This is likely due to the dispersal characteristics of the taxa investigated. Our data predominantly encapsulates insects with an aquatic larval stage (collected during kick-net) and a terrestrial winged adult stage (Ephemeroptera, Plecoptera, Trichoptera, and Diptera) (Additional file 1: Table S1). While it is hard to make generalization about the dispersal capabilities of such a diverse group of taxa, these organisms predominantly disperse using flight in their adult stage [64, 65], meaning they are not constrained to streams and can move between them over land. However, there is evidence that most adult insects stay within 50 m of stream corridors [66–69]. Additionally, in a mountain landscape, changes in elevation can act as a physical barrier even for flying insects [70]. Therefore, it is unsurprising that intraspecific structuring between region groups was observed. For a species, if dispersal between region groups remains infrequent, overtime species cohesion will be lost, further distinguishing species-level communities. Consistent patterns of community β-diversity and intraspecific genetic variation in DNA metabarcoding data were also observed in a metaphylogeographic analysis of marine barriers by Antich et al. [38]*.* This consistency is a reflection of microevolution giving rise to macroevolution [38, 39].

In our study, the species that had the strongest separation between region groups was *Y. brevis* (Fig. 6). Hughes et al. studied allozyme variation in *Y. brevis* from a valley in Montana [59]. They identified that the between streams there was higher variation than within streams; they suggest that the canyons separating these streams may be enough to impede the dispersal of weak fliers. These findings are generally consistent with our observations of strong region group separation for *Y. brevis*, although at a different geographic scale. As a consequence of this, Hughes et al. suggest recolonization by *Y. brevis* after a disturbance may be limited [59]. This presents a potential application for Biomonitoring 2.0 Refined, specifically investigating intraspecific structuring around barriers in DNA metabarcoding data.

Understanding the dispersal of organisms is essential for recolonization, a necessary step in habitat restoration [65, 70, 71]. If an area is being restored for a target group of organisms, it is essential that those organisms be able to reach that area from their current distribution [72, 73]. For example, if a species was known to exist on both sides of a mountain range, but is eradicated on one side by habitat disturbance. Its dispersal capabilities will inform if habitat restoration will be sufficient for recolonization to occur by individuals on the other side of the mountain range. Detecting intraspecific structuring using Biomonitoring 2.0 Refined could be useful for identifying where and what species are likely to have limited dispersal ability, and where recolonization efforts should be placed.

### Community β-diversity and intraspecific genetic variation are not readily compared

Comparing community β-diversity to intraspecific genetic variation presents its own challenges. One method we used was to test the significance of region group separation by genetic variation for each species proxy individually, similar to what Andújar et al. did to study habitat and spatial structuring on an oceanic island [42]. The portion of species proxies that had significant region group separation can then be measured (Table 1). While this does provide insight into how well individual species proxies are separated by region groups, there is no quantifiable way to compare this to community β-diversity and region group separation. The other method we used was to average all of the individual species proxy dissimilarity matrices into one matrix in order to get a general pattern of intraspecific genetic variation. This was similar to what Antich et al. did to study intraspecific genetic variation across marine barriers [38]. This method creates a single mean dissimilarity matrix that can be compared against the community dissimilarity matrix. However, community β-diversity was measured by comparing everything present in a site, while intraspecific genetic variation was measured by looking at smaller groups within each site and averaging these groups. This results in high dimensional comparisons of data for community β-diversity and a series of lower dimensional comparisons of data for intraspecific genetic variation. Due to the curse of dimensionality, specifically distance concentration, as the number of dimensions increases, objects in that space get further apart and can become difficult to meaningfully distinguish [74]. This could be causing the observed increase in dissimilarity apparent in the violin plots (Fig. 3C and Additional file 2: Fig. S7C), where the dissimilarities for the community ESVs are much closer to the maximum (1.0) than the intraspecific OTUs and SBCs.

To account for the difference in dimensionality, ESVs in OTUs and SBCs were scrambled to create new clusters that maintain the same structure (the same number of clusters each with the same number of ESVs, Additional file 2: Fig. S4) but are not representative of species. The β-diversity observed within the clusters should reflect samplings of the haplotype-level community, because the ESVs have no defined relationship in the clusters. This enabled the creation of mean dissimilarity matrices that were representative of community β-diversity. Scrambled OTUs and SBCs resulted in pseudo *F*-statistics of region group separation that were closer to the intraspecific OTUs and SBCs (Fig. 4), suggesting that this comparison may be more equivalent than using the community ESVs. Interestingly, in the northeast and southeast region groups, there was significant spatial structuring for the scrambled OTUs and SBCs; however, the intraspecific OTUs and SBCs either lacked significant or had weaker spatial structuring (Fig. 5). This could suggest that species in this analysis that are spread across multiple region groups can freely disperse within the northeast and southeast region groups, even though the communities within these regions are distinct enough for there to be significant spatial structuring. The scope of this study was limited to comparing sampling locations in 2-dimensional space and indirectly separating regions based on elevation. Features, such as habitat, elevation, and anthropogenic factors, could provide greater insight into why and where community β-diversity and intraspecific genetic variation differ. If Biomonitoring 2.0 Refined provides a finer-scale analysis for biodiversity, then it will be important to consider site features on a finer-scale as well.

### Multiple amplicon validation

When using metabarcoding to study intraspecific genetic variation, a great deal of emphasis is placed on ensuring erroneous sequences are removed [41, 52]. This is most commonly done by removing sequences with low abundance and grouping sequences by similarity [56, 75, 76]. Errors can occur during the PCR, indexing, and sequencing steps [53, 77–79]. When investigating intraspecific genetic variation, single nucleotide differences maybe be used to distinguish between individuals [38, 41, 42, 52]. When a sequence variant is rare, it is assumed that this reflects PCR or sequencing error. If more copies of a sequence are present, then there is more confidence that this sequence variant is real, not just an artifact. This is because genuine DNA templates have more chances for exponential amplification during PCR, and therefore, should have many sequence copies after sequencing. A factor that contributes to the number of sequences present include the initial template abundance in the sample. Fundamentally, bioinformatic filter parameter choice comes down to a balance between removing rare and erroneous sequences [37, 43, 76]. The filters used in this study were intended to be strict with the goal of removing erroneous sequences and accepting the risk of removing genuine but rare sequences. Additionally, species were identified more rigorously by using SBCs that formed distinct clusters. If sequences truly belong to the same species, it would be expected that sequences would be close together in sequence space and isolated from sequences assigned to other species. By defining species this way, sequences that are erroneously grouped into species may be avoided except in cases where the chosen DNA marker region is too conserved and lacks the sequence variation needed to distinguish between species or if closely related species are erroneously assigned to the same species.

To help validate results, multiple amplicons targeting COI were compared. If multiple amplicons are used, results can be validated by looking for patterns that are consistent across amplicons [80]. Previous studies have used multiple markers in DNA to study the intraspecific diversity of targeted species [81, 82] and Elbrecht et al. compared multiple amplicons from broadly targeted DNA metabarcoding to identify consistent patterns within species [52]. However, the amplicons they were using overlap, meaning that some of the consistent patterns may be arising from the same sequence differences. If non-overlapping amplicons are compared, then the observed patterns of diversity should be consistent with the amplicons coming from the same gene, but independent with differences between amplicons coming from different nucleotide changes. The amplicons used in this study, F230R and MLJG, do not overlap, and all results previously discussed were consistently observed between them. Additionally, in *Y. brevis*, we were able to identify that F230R and MLJG ESVs that occurred in the same region groups were identified in the same barcodes (Fig. 7 and Additional file 2: Fig. S8). This consistency supports both how intraspecific diversity was investigated and the filtering parameters used. However, patterns observed using MLJG were always weaker based on pseudo *F*-statistics (Additional file 1: Tables S2 and S5). While this may be influenced by primer biases, the choice of filter parameters may be impacting MLJG more than F230R. MLJG has more ESVs than F230R before abundance filtering, but fewer after. MLJG is a longer sequence and therefore has greater sequence variation potential, which can result in fewer reads and more ESVs filtered with strict parameters. In future studies, it may be beneficial to optimize filter parameters for each individual primer.

## Conclusions

In this work, we present the idea of Biomonitoring 2.0 Refined through the application of community sampling coupled with multi-amplicon metabarcoding and statistical analyses. Our work clearly demonstrates that, in the Rocky Mountains, DNA metabarcoding can be used to identify and validate intraspecific diversity structuring in aquatic arthropods. Similar structuring was also observed at the community level; this is likely due to the strong affect mountains have as a barrier to dispersal. However, within northeast and southeast regions, community and intraspecific diversity had distinct structuring. In order to further develop Biomonitoring 2.0 Refined, a finer-scale of site features should also be considered to capture the potential sensitivity of intraspecific diversity. Overall, this study sets the stage for utilizing Biomonitoring 2.0 Refined in a wider context for understanding biodiversity change and for applications in conservation planning, nature impact studies, and environmental stewardship.

## Methods

### Sample collection, processing, and selection

All samples were collected and processed as a part of the community-based project Sequencing The Rivers for Environmental Assessment and Monitoring (STREAM) [22]. Benthic samples were collected using the kick-net method described in STREAM’s Field Manual. Briefly, at each site three kick-nets are performed; each subsequent replicate is done further upstream to sample as many microhabitats as possible and to avoid previously disturbed areas. After performing kick-net, the entire contents (whole organisms, sediment, vegetation, etc.) of the net is transferred to a sample jar that is subsequently filled with a preservative (antifreeze or ethanol). Rocks and observed living vertebrates are removed from the net, because rocks are a hazard during homogenization and vertebrates are not the target of these analyses. Samples were processed using the STREAM Benthic Metabarcoding Lab Protocol v2 [83]. Briefly, samples were homogenized using blenders and incubated at 70 °C to remove preservative (antifreeze or ethanol). Dried samples were used with Qiagen PowerSoil Pro kit to extract DNA. Samples were amplified using a two-step polymerase chain reaction (PCR) targeting three regions in the COI DNA barcode region. The primer sets used were B/ArR5 (∼310 bp) referred to as BR5, LCO1490/230_R (∼230 bp) referred to as F230R, and mICOIintF/jgHCO2198 (∼313 bp) referred to as MLJG [84–87]. Sequencing was performed using Illumina MiSeq with a MiSeq Reagent Kit v3 (paired-ends 2 × 300 bp).

From the STREAM project, a total of 138 samples were selected; all samples were collected in triplicate and were gathered in 2019, 2020, 2021, or 2022. All sample sites were located between longitude − 118 to − 113 and latitude 49 to 52. This area covers the eastern edge of the Rocky Mountains between British Columbia and Alberta. In one sample, MLJG only had sequences in one replicate; this sample was omitted for analyses performed on MLJG (X2021_50.89445_116.35212). This also resulted in the removal of 13 MLJG sequences only found in this replicate after sequence processing.

All analyses in R were preformed using version 4.2.1 [88]. Samples were grouped by location (latitude and longitude) with k-means clustering using the kmeans function from the stats package in R. Samples were clustered with 2 to 20 cluster centers. For each cluster center value, two clusterings were performed and the Rand index between the clusterings was calculated using the randIndex function from the flexclust package [89]. Each comparison of two clusters was performed 1000 times to identify the number of cluster centers that gave the most stable clusters. Additionally, the within-cluster sum of squares was calculated and extracted using the purrr R package [90]. The clusters obtained are referred to as region groups.

Sampling location maps were created using the R packages: ggplot2 [91], raster [92], elevatr [93], sf [94, 95], tidyterra [96], ggnewscale [97], rnaturalearth [98], rnaturalearthdata [99], cowplot [100], and scatterpie [101].

### Bioinformatic sequence processing

Raw sequences were run through the MetaWorks 1.13.0 pipeline using the default settings in the ESV workflow for COI [55]. Briefly, MetaWorks merged raw paired-end reads using SEQPREP v1.3.2 [102]; this was followed by primer trimming using CUTADAPT v4.1 [103]. Sequences were dereplicated, denoised, and putative chimeric sequences were removed using fastx_unique, unoise3, and uchime3_denovo, respectively, from VSEARCH v2.21.1 [104]. An *α* = 2.0 was used for denoising and only clusters with a minimum of 8 reads were preserved. Taxonomic assignment was performed using the Ribosomal Database Project (RDP) classifier for Eukaryote COI mtDNA sequences (RDP COI v5.1.0) [105, 106]. After taxonomic assignment, only sequences assigned to Arthropoda were retained. Finally, putative pseudogenes were removed by comparing a profile hidden Markov model (HMM) of the sequences to a profile HMM created using BOLD arthropod COI barcode sequences [107]. The sequence profile HMM was generated by translating sequences using ORFinder v0.4.3 [108] and running a profile HMM analysis using HMMER v3.3.2 [109].

Data organization and preparation was performed using Python 3.9.2 [110]. Exact sequence variants (ESVs) were separated by amplicon (BR5 ESVs were omitted from further analyses, because BR5 overlaps with both F230R and MLJG) and an additional multiple sequence alignment filtering step was performed. Because COI is a protein coding gene, we expected high-quality COI sequences to align without gaps to preserve codon structure [111]. Multiple sequence alignment filtering was used to filter out sequences with putative insertions/deletions (indels) causing frame shifts. The gap opening score was set to 9999 (very high), so that gaps could only be placed at the start or end of sequences. If sequences have either a leading gap and/or unaligned trailing sequence, these sequences were removed as being putative artifacts (pseudogenes or the result of PCR/sequencing error) (Fig. S1). Because genuine COI sequences should be represented by many sequences following PCR amplification, following MSA filtering, ESVs with less than 100 reads were removed as a further quality control step. Finally, ESVs were removed from a sample replicate if there were fewer than 5 reads present to avoid tag-switching between samples. Absolute read count filters were selected for readability and reproducibility. Sample replicates were pooled and read counts were converted into presence/absence.

### Community β-diversity

Community β-diversity was assessed using three different methods: as individual ESVs, ESVs merged into OTUs, and ESVs merged into species bound clusters (SBCs). ESVs were clustered into OTUs using SWARM 3.1.3 with the no OTU breaking (-n) option enabled; *d* (maximum number of differences) was selected based on the value that was closest to 3% of the amplicon length [112]. For F230R, a *d* of 7 was used (7 nt/229 nt × 100% = 3.06%) and a *d* of 9 was used for MLJG (9 nt/313 nt × 100% = 2.88). SBCs were also obtained by clustering ESVs using SWARM 3.1.3 with the no OTU breaking (-n) option enabled [112]; this was to ensure that at varying thresholds clusters maintained a hierarchal structure. Using a Python script, clustering was started at a threshold of *d* = 0 and *d* was increased by one until all ESVs were in a single cluster. ESVs were assigned to a species if their species-level bootstrap value (sBP) from the RDP classifier was equal to or greater than a threshold of 0.8 (≥ 95% correct assignment if query sequences are present) [106]. ESVs with bootstrap values below the selected threshold were considered unassigned. For each assigned species, clusters were identified that included all of that species assigned ESVs. If a cluster only contained its assigned ESVs, it was considered to be an SBC (it could also contain unassigned ESVs). For each SBC, the lowest *d* value and the highest *d* value in which the cluster was present were recorded. For community representations, ESVs in OTUs and SBCs were merged to get presence/absence tables of OTUs and SBCs in samples (Fig. S2). Sørensen dissimilarity matrices were calculated using the beta.pair.abund function from the betapart R package on the presence/absence tables (equivalent to beta.pair function) [113].

### Intraspecific genetic diversity

After OTUs and SBCs were obtained, clusters that had 2 or more haplotypes and were present in at least 2 region groups in at least 3 samples per region group (present in a minimum of 6 samples) were selected for intraspecific analysis. For each cluster, a dissimilarity matrix was created also using Sørensen dissimilarity. Cluster dissimilarity matrices were combined by calculating the mean dissimilarity for each pair of samples across all species (Fig. S3) [38]. If there were pairs of samples that had no dissimilarities calculated, the sample that had the most missing pairs was removed. This was done until the matrix had no missing dissimilarities between samples. Only samples present in both intraspecific OTUs and SBCs were compared.

### Analysis of dissimilarity and region groups

To determine if dissimilarity was significantly different between region groups, permutational analysis of variance (PERMANOVA) was performed on the dissimilarity matrices using the region groups as grouping factors with the adonis2 function from the vegan R package with 9999 permutations [114]. Significance was determined by a *p* value < 0.05. In cases where multiple tests were performed, *p* values were adjusted using the false discovery rate (FDR) method. Dissimilarity dispersion within region groups was calculated using the betadisper function from the vegan R package [114]. To test if dispersion was significantly different between region groups, the permutest function from the vegan R package was used with 9999 permutations [114]. Finally, ordination of dissimilarity matrices was performed using uniform manifold approximation and projection (UMAP) with the umap function from the uwot R package using default settings unless otherwise specified [115].

### Scrambled clusters

ESVs within OTUs and SBCs were shuffled to create scrambled clusters representing OTUs and SBCs (Fig. S4). Scrambled clusters were created 1000 times for both OTUs and SBCs and mean dissimilarity matrices were created using the same method to create intraspecific OTUs and SBCs. For consistency, only sites that overlapped with sites in the intraspecific OTUs and SBCs were analyzed.

### Geographic distances

The geodesic distance was calculated between sampling locations to account for the spherical nature of the Earth. The distance was calculated using World Geodetic System 1984 (WGS84) coordinates and the distm and distGeo functions from the geosphere R package [116].

### Matrix correlations

For all matrix correlations, the Spearman’s correlation between matrices was calculated. Spearmen’s correlation is 1 if a relationship between two variables is monotonic, both variables increase together, and is − 1 when one variable increases the other variable always decreases. The correlation and significance were calculated using the Mantel test using the mantel function with 9999 permutations from the vegan R package [114].

## Supplementary Information

Additional file 1: Tables S1–S11. Table S1 Number of species bound clusters belonging to each order. Table S2 Permutational analysis of variance of community representations on region group separation using Sørensen dissimilarity. Table S3 Dispersion comparison of community representations on region group separation using Sørensen dissimilarity.Table S4 Pairwise permutational analysis of variance of community representations on region group separation using Sørensen dissimilarity. Table S5 Permutational analysis of variance of intraspecific and community representations on region group separation using Sørensen dissimilarity. Table S6 Dispersion comparison of intraspecific and community representations on region group separation using Sørensen dissimilarity. Table S7 Pairwise permutational analysis of variance of intraspecific and community representations on region group separation using Sørensen dissimilarity. Table S8 Spearman’s rank correlation between intraspecific genetic variation and community β-diversity dissimilarity matrices. Table S9 Spearman’s rank correlation between intraspecific genetic variation and geodesic distances of sampling location. Table S10 Summary of Spearman’s rank correlation between scrambled cluster β-diversity and geodesic distances of sampling location. Table S11 Comparison of SBCs with F230R and MLJG amplicons.

Additional file 2: Figures S1–S8. Fig. S1 Schematic of multiple sequence alignment filtering. Fig. S2 Schematic of ESV merging by cluster and dissimilarity matrix generation. Fig. S3 Schematic of ESV grouping by cluster and mean dissimilarity matrix generation. Fig. S4 Schematic of ESV scrambling by cluster to generate scrambled clusters. Fig. S5 Comparisons of adjusted Rand index using different numbers of cluster centers to separate sites into region groups. Fig. S6 Comparisons of total within-cluster sum of squares using different numbers of cluster centers to separate sites into region groups. Fig. S7 Intraspecific genetic variation separates region groups with dissimilarity patterns that differ from community β-diversity (MLJG). Fig. S8 Multiple sequence alignment of *Yoraperla brevis *ESVs and barcodes.

## Data Availability

Sequence data that supports the findings of this study have been deposited in the Sequence Read Archive with the primary accession code PRJNA1201794 [117]. Code for processing data and generating figures and tables is available at github.com/anriley/Biomonitoring-2.0-Refined [118].
