# Supplementary material for: Biomonitoring 2.0 Refined: observing local change through metaphylogeography using a community-based eDNA metabarcoding monitoring network
Source: BMC Biol. 2025 Jul 1;23:187. doi: 10.1186/s12915-025-02284-x (PMC12220750; doi:10.1186/s12915-025-02284-x)
Supplement: Supplementary file 1 — Additional file 1: Tables S1–S11. Table S1 Number of species bound clusters belonging to each order. Table S2 Permutational analysis of variance of community representations on region group separation using Sørensen dissimilarity. Table S3 Dispersion comparison of community representations on region group separation using Sørensen dissimilarity.Table S4 Pairwise permutational analysis of variance of community representations on region group separation using Sørensen dissimilarity. Table S5 Permutational analysis of variance of intraspecific and community representations on region group separation using Sørensen dissimilarity. Table S6 Dispersion comparison of intraspecific and community representations on region group separation using Sørensen dissimilarity. Table S7 Pairwise permutational analysis of variance of intraspecific and community representations on region group separation using Sørensen dissimilarity. Table S8 Spearman’s rank correlation between intraspecific genetic variation and community β-diversity dissimilarity matrices. Table S9 Spearman’s rank correlation between intraspecific genetic variation and geodesic distances of sampling location. Table S10 Summary of Spearman’s rank correlation between scrambled cluster β-diversity and geodesic distances of sampling location. Table S11 Comparison of SBCs with F230R and MLJG amplicons. [file 12915_2025_2284_MOESM1_ESM.docx]

**Table S1. Number of species bound clusters belonging to each order.**

| **Order** | **F230R** | **MLJG** |
| --- | --- | --- |
| **Amphipoda** | 2 | 1 |
| **Araneae** | 3 | 5 |
| **Calanoida** | 1 | 0 |
| **Coleoptera** | 13 | 7 |
| **Cyclopoida** | 1 | 0 |
| **Decapoda** | 1 | 0 |
| **Diplostraca** | 7 | 8 |
| **Diptera** | 154 | 75 |
| **Ephemeroptera** | 59 | 39 |
| **Hemiptera** | 20 | 9 |
| **Hymenoptera** | 9 | 4 |
| **Isopoda** | 1 | 0 |
| **Julida** | 2 | 2 |
| **Lepidoptera** | 12 | 4 |
| **Odonata** | 8 | 5 |
| **Opiliones** | 0 | 2 |
| **Orthoptera** | 2 | 0 |
| **Plecoptera** | 49 | 34 |
| **Podocopida** | 2 | 2 |
| **Sarcoptiformes** | 8 | 2 |
| **Symphypleona** | 1 | 0 |
| **Trichoptera** | 56 | 37 |
| **Trombidiformes** | 0 | 2 |

**Table S2. Permutational analysis of variance of community representations on region group separation using Sørensen dissimilarity.**

|  | | | **Df** | **SumOfSqs** | **R2** | **F** | **Pr(>F)** |
| --- | --- | --- | --- | --- | --- | --- | --- |
| **F230R** | Community  ESVs | Region group | 3 | 8.198912 | 0.204461 | 10.36607 | 1.00E-04 |
|  |  | Residual | 121 | 31.90113 | 0.795539 | NA | NA |
|  |  | Total | 124 | 40.10005 | 1 | NA | NA |
|  | Community OTUs | Region group | 3 | 6.118244 | 0.19265 | 9.624364 | 1.00E-04 |
|  |  | Residual | 121 | 25.64005 | 0.80735 | NA | NA |
|  |  | Total | 124 | 31.75829 | 1 | NA | NA |
|  | Community SBCs | Region group | 3 | 4.751389 | 0.18074 | 8.89811 | 1.00E-04 |
|  |  | Residual | 121 | 21.53709 | 0.81926 | NA | NA |
|  |  | Total | 124 | 26.28847 | 1 | NA | NA |
| **MLJG** | Community ESVs | Region group | 3 | 7.936088 | 0.177751 | 8.647081 | 1.00E-04 |
|  |  | Residual | 120 | 36.71106 | 0.822249 | NA | NA |
|  |  | Total | 123 | 44.64715 | 1 | NA | NA |
|  | Community OTUs | Region group | 3 | 6.597711 | 0.166137 | 7.969507 | 1.00E-04 |
|  |  | Residual | 120 | 33.11477 | 0.833863 | NA | NA |
|  |  | Total | 123 | 39.71249 | 1 | NA | NA |
|  | Community SBCs | Region group | 3 | 5.876119 | 0.175543 | 8.516809 | 1.00E-04 |
|  |  | Residual | 120 | 27.59775 | 0.824457 | NA | NA |
|  |  | Total | 123 | 33.47387 | 1 | NA | NA |

Abbreviations: ESV, exact sequence variant; OTU, operational taxonomic unit; SBC, species bound cluster.

**Table S3. Dispersion comparison of community representations on region group separation using Sørensen dissimilarity.**

|  | | | **Df** | **Sum Sq** | **Mean Sq** | **F** | **N.Perm** | **Pr(>F)** |
| --- | --- | --- | --- | --- | --- | --- | --- | --- |
| **F230R** | Community ESVs | Groups | 3 | 0.078241 | 0.02608 | 2.191463 | 9999 | 0.0872 |
|  |  | Residuals | 121 | 1.440012 | 0.011901 | NA | NA | NA |
|  | Community OTUs | Groups | 3 | 0.094384 | 0.031461 | 2.214347 | 9999 | 0.0832 |
|  |  | Residuals | 121 | 1.719154 | 0.014208 | NA | NA | NA |
|  | Community SBCs | Groups | 3 | 0.119115 | 0.039705 | 2.288842 | 9999 | 0.08 |
|  |  | Residuals | 121 | 2.099003 | 0.017347 | NA | NA | NA |
| **MLJG** | Community ESVs | Groups | 3 | 0.038354 | 0.012785 | 1.036106 | 9999 | 0.3721 |
|  |  | Residuals | 120 | 1.480714 | 0.012339 | NA | NA | NA |
|  | Community OTUs | Groups | 3 | 0.04067 | 0.013557 | 1.18848 | 9999 | 0.3098 |
|  |  | Residuals | 120 | 1.368821 | 0.011407 | NA | NA | NA |
|  | Community SBCs | Groups | 3 | 0.114393 | 0.038131 | 1.789482 | 9999 | 0.1577 |
|  |  | Residuals | 120 | 2.556999 | 0.021308 | NA | NA | NA |

Abbreviations: ESV, exact sequence variant; OTU, operational taxonomic unit; SBC, species bound cluster.

**Table S4. Pairwise permutational analysis of variance of community representations on region group separation using Sørensen dissimilarity.**

|  | | **Region pairs** | **F** | **R2** | **Pr(>F)** | **Pr(>F) adjusted** |
| --- | --- | --- | --- | --- | --- | --- |
| **F230R** | Community ESVs | Central Northeast | 10.73318 | 0.153918 | 1.00E-04 | 1.00E-04 |
|  |  | Central Southeast | 8.744437 | 0.120208 | 1.00E-04 | 1.00E-04 |
|  |  | Central West | 12.65907 | 0.156945 | 1.00E-04 | 1.00E-04 |
|  |  | Northeast Southeast | 5.905735 | 0.100257 | 1.00E-04 | 1.00E-04 |
|  |  | Northeast West | 14.15317 | 0.198911 | 1.00E-04 | 1.00E-04 |
|  |  | Southeast West | 10.31016 | 0.142582 | 1.00E-04 | 1.00E-04 |
|  | Community OTUs | Central Northeast | 11.34329 | 0.161256 | 1.00E-04 | 1.00E-04 |
|  |  | Central Southeast | 9.255193 | 0.126342 | 1.00E-04 | 1.00E-04 |
|  |  | Central West | 11.9772 | 0.149758 | 1.00E-04 | 1.00E-04 |
|  |  | Northeast Southeast | 5.152613 | 0.088605 | 1.00E-04 | 1.00E-04 |
|  |  | Northeast West | 12.54512 | 0.180388 | 1.00E-04 | 1.00E-04 |
|  |  | Southeast West | 7.889704 | 0.112888 | 1.00E-04 | 1.00E-04 |
|  | Community SBCs | Central Northeast | 12.33353 | 0.1729 | 1.00E-04 | 1.00E-04 |
|  |  | Central Southeast | 9.12856 | 0.124829 | 1.00E-04 | 1.00E-04 |
|  |  | Central West | 9.308524 | 0.120407 | 1.00E-04 | 1.00E-04 |
|  |  | Northeast Southeast | 5.61614 | 0.095812 | 1.00E-04 | 1.00E-04 |
|  |  | Northeast West | 11.41723 | 0.166877 | 1.00E-04 | 1.00E-04 |
|  |  | Southeast West | 6.436739 | 0.094054 | 1.00E-04 | 1.00E-04 |
| **MLJG** | Community ESVs | Central Northeast | 7.324132 | 0.11212 | 1.00E-04 | 1.00E-04 |
|  |  | Central Southeast | 8.25624 | 0.115867 | 1.00E-04 | 1.00E-04 |
|  |  | Central West | 10.3833 | 0.13418 | 1.00E-04 | 1.00E-04 |
|  |  | Northeast Southeast | 5.40057 | 0.092475 | 1.00E-04 | 1.00E-04 |
|  |  | Northeast West | 10.77076 | 0.158929 | 1.00E-04 | 1.00E-04 |
|  |  | Southeast West | 9.536171 | 0.133306 | 1.00E-04 | 1.00E-04 |
|  | Community OTUs | Central Northeast | 7.329353 | 0.112191 | 1.00E-04 | 1.00E-04 |
|  |  | Central Southeast | 7.098133 | 0.10126 | 1.00E-04 | 1.00E-04 |
|  |  | Central West | 9.26967 | 0.121538 | 1.00E-04 | 1.00E-04 |
|  |  | Northeast Southeast | 4.915403 | 0.084872 | 1.00E-04 | 1.00E-04 |
|  |  | Northeast West | 10.65102 | 0.157441 | 1.00E-04 | 1.00E-04 |
|  |  | Southeast West | 8.613044 | 0.121975 | 1.00E-04 | 1.00E-04 |
|  | Community SBCs | Central Northeast | 11.16124 | 0.16138 | 1.00E-04 | 1.00E-04 |
|  |  | Central Southeast | 7.899437 | 0.111417 | 1.00E-04 | 1.00E-04 |
|  |  | Central West | 8.707406 | 0.115014 | 1.00E-04 | 1.00E-04 |
|  |  | Northeast Southeast | 4.550556 | 0.079071 | 1.00E-04 | 1.00E-04 |
|  |  | Northeast West | 11.48409 | 0.16769 | 1.00E-04 | 1.00E-04 |
|  |  | Southeast West | 7.838096 | 0.112232 | 1.00E-04 | 1.00E-04 |

Adjusted p-values were calculated using the false discovery rate method. Abbreviations: ESV, exact sequence variant; OTU, operational taxonomic unit; SBC, species bound cluster.

**Table S5. Permutational analysis of variance of population and community representations on region group separation using Sørensen dissimilarity.**

|  | | | **Df** | **SumOfSqs** | **R2** | **F** | **Pr(>F)** |
| --- | --- | --- | --- | --- | --- | --- | --- |
| **F230R** | Community ESVs | Region group | 3 | 8.129489 | 0.215037 | 10.68388 | 1.00E-04 |
|  |  | Residual | 117 | 29.67555 | 0.784963 | NA | NA |
|  |  | Total | 120 | 37.80504 | 1 | NA | NA |
|  | Intraspecific OTUs | Region group | 3 | 3.542564 | 0.386961 | 24.61747 | 1.00E-04 |
|  |  | Residual | 117 | 5.612274 | 0.613039 | NA | NA |
|  |  | Total | 120 | 9.154837 | 1 | NA | NA |
|  | Intraspecific SBCs | Region group | 3 | 3.244087 | 0.398202 | 25.80576 | 1.00E-04 |
|  |  | Residual | 117 | 4.902757 | 0.601798 | NA | NA |
|  |  | Total | 120 | 8.146843 | 1 | NA | NA |
| **MLJG** | Community ESVs | Region group | 3 | 7.473447 | 0.224086 | 9.337955 | 1.00E-04 |
|  |  | Residual | 97 | 25.87734 | 0.775914 | NA | NA |
|  |  | Total | 100 | 33.35079 | 1 | NA | NA |
|  | Intraspecific OTUs | Region group | 3 | 3.19709 | 0.37833 | 19.67707 | 1.00E-04 |
|  |  | Residual | 97 | 5.253453 | 0.62167 | NA | NA |
|  |  | Total | 100 | 8.450543 | 1 | NA | NA |
|  | Intraspecific SBCs | Region group | 3 | 2.193528 | 0.291833 | 13.32443 | 1.00E-04 |
|  |  | Residual | 97 | 5.32286 | 0.708167 | NA | NA |
|  |  | Total | 100 | 7.516388 | 1 | NA | NA |

Abbreviations: ESV, exact sequence variant; OTU, operational taxonomic unit; SBC, species bound cluster.

**Table S6. Dispersion comparison of population and community representations on region group separation using Sørensen dissimilarity.**

|  | |  | **Df** | **Sum Sq** | **Mean Sq** | **F** | **N.Perm** | **Pr(>F)** |
| --- | --- | --- | --- | --- | --- | --- | --- | --- |
| **F230R** | Community ESVs | Groups | 3 | 0.014813 | 0.004938 | 1.117731 | 9999 | 0.3378 |
|  |  | Residuals | 117 | 0.516871 | 0.004418 | NA | NA | NA |
|  | Intraspecific OTUs | Groups | 3 | 0.011848 | 0.003949 | 0.997025 | 9999 | 0.396 |
|  |  | Residuals | 117 | 0.463451 | 0.003961 | NA | NA | NA |
|  | Intraspecific SBCs | Groups | 3 | 0.019092 | 0.006364 | 0.591343 | 9999 | 0.6128 |
|  |  | Residuals | 117 | 1.259167 | 0.010762 | NA | NA | NA |
| **MLJG** | Community ESVs | Groups | 3 | 0.070352 | 0.023451 | 2.679818 | 9999 | 0.0527 |
|  |  | Residuals | 97 | 0.848831 | 0.008751 | NA | NA | NA |
|  | Intraspecific OTUs | Groups | 3 | 0.01622 | 0.005407 | 0.747794 | 9999 | 0.5308 |
|  |  | Residuals | 97 | 0.70132 | 0.00723 | NA | NA | NA |
|  | Intraspecific SBCs | Groups | 3 | 0.010454 | 0.003485 | 0.325935 | 9999 | 0.8072 |
|  |  | Residuals | 97 | 1.037029 | 0.010691 | NA | NA | NA |

Abbreviations: ESV, exact sequence variant; OTU, operational taxonomic unit; SBC, species bound cluster.

**Table S7. Pairwise permutational analysis of variance of intraspecific and community representations on region group separation using Sørensen dissimilarity.**

|  | | **Region pairs** | **F** | **R2** | **Pr(>F)** | **Pr(>F) adjusted** |
| --- | --- | --- | --- | --- | --- | --- |
| **F230R** | Community ESVs | Central Northeast | 10.73318 | 0.153918 | 1.00E-04 | 1.00E-04 |
|  |  | Central Southeast | 9.000061 | 0.128572 | 1.00E-04 | 1.00E-04 |
|  |  | Central West | 12.49501 | 0.15718 | 1.00E-04 | 1.00E-04 |
|  |  | Northeast Southeast | 6.535145 | 0.115594 | 1.00E-04 | 1.00E-04 |
|  |  | Northeast West | 14.19566 | 0.20223 | 1.00E-04 | 1.00E-04 |
|  |  | Southeast West | 10.88028 | 0.157959 | 1.00E-04 | 1.00E-04 |
|  | Intraspecific OTUs | Central Northeast | 19.16846 | 0.24522 | 1.00E-04 | 1.00E-04 |
|  |  | Central Southeast | 13.26367 | 0.178602 | 1.00E-04 | 1.00E-04 |
|  |  | Central West | 24.90477 | 0.270985 | 1.00E-04 | 1.00E-04 |
|  |  | Northeast Southeast | 12.58637 | 0.201104 | 1.00E-04 | 1.00E-04 |
|  |  | Northeast West | 42.38266 | 0.430794 | 1.00E-04 | 1.00E-04 |
|  |  | Southeast West | 38.1844 | 0.396992 | 1.00E-04 | 1.00E-04 |
|  | Intraspecific SBCs | Central Northeast | 20.30033 | 0.255993 | 1.00E-04 | 1.00E-04 |
|  |  | Central Southeast | 17.30922 | 0.221037 | 1.00E-04 | 1.00E-04 |
|  |  | Central West | 28.48764 | 0.298339 | 1.00E-04 | 1.00E-04 |
|  |  | Northeast Southeast | 14.34562 | 0.222946 | 1.00E-04 | 1.00E-04 |
|  |  | Northeast West | 32.80447 | 0.369401 | 1.00E-04 | 1.00E-04 |
|  |  | Southeast West | 40.47217 | 0.411001 | 1.00E-04 | 1.00E-04 |
| **MLJG** | Community ESVs | Central Northeast | 7.101075 | 0.133728 | 1.00E-04 | 1.00E-04 |
|  |  | Central Southeast | 8.582429 | 0.146502 | 1.00E-04 | 1.00E-04 |
|  |  | Central West | 11.71723 | 0.165691 | 1.00E-04 | 1.00E-04 |
|  |  | Northeast Southeast | 5.859171 | 0.133591 | 1.00E-04 | 1.00E-04 |
|  |  | Northeast West | 11.1893 | 0.192291 | 1.00E-04 | 1.00E-04 |
|  |  | Southeast West | 10.33096 | 0.168446 | 1.00E-04 | 1.00E-04 |
|  | Intraspecific OTUs | Central Northeast | 13.24615 | 0.223578 | 1.00E-04 | 1.00E-04 |
|  |  | Central Southeast | 11.40695 | 0.18576 | 1.00E-04 | 1.00E-04 |
|  |  | Central West | 25.04452 | 0.297991 | 1.00E-04 | 1.00E-04 |
|  |  | Northeast Southeast | 7.817451 | 0.170622 | 1.00E-04 | 1.00E-04 |
|  |  | Northeast West | 34.67385 | 0.42454 | 1.00E-04 | 1.00E-04 |
|  |  | Southeast West | 24.12425 | 0.321125 | 1.00E-04 | 1.00E-04 |
|  | Intraspecific SBCs | Central Northeast | 16.23838 | 0.260906 | 1.00E-04 | 0.00012 |
|  |  | Central Southeast | 12.50446 | 0.200057 | 1.00E-04 | 0.00012 |
|  |  | Central West | 9.414192 | 0.137606 | 1.00E-04 | 0.00012 |
|  |  | Northeast Southeast | 5.190635 | 0.12018 | 1.00E-04 | 2.00E-04 |
|  |  | Northeast West | 21.91915 | 0.318041 | 1.00E-04 | 0.00012 |
|  |  | Southeast West | 14.21273 | 0.217944 | 1.00E-04 | 0.00012 |

Adjusted p-values were calculated using the false discovery rate method. Abbreviations: ESV, exact sequence variant; OTU, operational taxonomic unit; SBC, species bound cluster.

**Table S8. Spearman’s rank correlation between intraspecific genetic variation and community β-diversity dissimilarity matrices.**

| **Dissimilarity matrices** | | **Spearman's ρ** | **p-value** |
| --- | --- | --- | --- |
| F230R  Intraspecific SBCs | F230R  Intraspecific OTUs | 0.736132 | 1.00E-04 |
| F230R  Intraspecific SBCs | F230R  Community ESVs | 0.304938 | 1.00E-04 |
| F230R  Intraspecific OTUs | F230R  Community ESVs | 0.441129 | 1.00E-04 |
| MLJG  Intraspecific SBCs | MLJG  Intraspecific OTUs | 0.630882 | 1.00E-04 |
| MLJG  Intraspecific SBCs | MLJG  Community ESVs | 0.275503 | 1.00E-04 |
| MLJG  Intraspecific OTUs | MLJG  Community ESVs | 0.427222 | 1.00E-04 |
| F230R  Intraspecific SBCs | MLJG  Intraspecific SBCs | 0.276548 | 1.00E-04 |
| F230R  Intraspecific OTUs | MLJG  Intraspecific OTUs | 0.405068 | 1.00E-04 |

Abbreviations: ESV, exact sequence variant; OTU, operational taxonomic unit; SBC, species bound cluster.

**Table S9. Spearman’s rank correlation between intraspecific genetic variation and geodesic distances of sampling location.**

|  | | **Region** | **correlation** | **p-value** |
| --- | --- | --- | --- | --- |
| **F230R** | Intraspecific OTUs | Northeast | 0.28217868 | 0.0078 |
|  |  | Southeast | 0.269095316 | 2.00E-04 |
|  |  | Central | 0.372383995 | 1.00E-04 |
|  |  | West | 0.379235436 | 2.00E-04 |
|  | Intraspecific SBCs | Northeast | 0.095096373 | 0.1705 |
|  |  | Southeast | 0.216548595 | 0.0012 |
|  |  | Central | 0.30286019 | 1.00E-04 |
|  |  | West | 0.357595265 | 5.00E-04 |
| **MLJG** | Intraspecific OTUs | Northeast | 0.100186464 | 0.1655 |
|  |  | Southeast | 0.126906303 | 0.057 |
|  |  | Central | 0.4232798 | 1.00E-04 |
|  |  | West | 0.266064392 | 0.0063 |
|  | Intraspecific SBCs | Northeast | -0.043305339 | 0.6682 |
|  |  | Southeast | 0.041957257 | 0.3138 |
|  |  | Central | 0.301533902 | 1.00E-04 |
|  |  | West | 0.292793234 | 0.0013 |

Abbreviations: ESV, exact sequence variant; OTU, operational taxonomic unit; SBC, species bound cluster.

**Table S10. Summary of Spearman’s rank correlation between scrambled clusters β-diversity and geodesic distances of sampling location.** Significance was determined with a *p*-value < 0.05.

|  | | **Region** | **Mean ρ** | **Standard deviation** | **Min** | **Q1** | **Median** | **Q3** | **Max** | **Significant ρ** |
| --- | --- | --- | --- | --- | --- | --- | --- | --- | --- | --- |
| **F230R** | Scrambled OTUs | Northeast | 0.6246 | 0.0487 | 0.4610 | 0.5937 | 0.6289 | 0.6586 | 0.7789 | 1000 |
|  |  | Southeast | 0.4978 | 0.0390 | 0.3503 | 0.4717 | 0.4974 | 0.5251 | 0.6075 | 1000 |
|  |  | Central | 0.2718 | 0.0451 | 0.1233 | 0.2430 | 0.2746 | 0.3039 | 0.3943 | 1000 |
|  |  | West | 0.3415 | 0.0453 | 0.1962 | 0.3129 | 0.3437 | 0.3708 | 0.4678 | 1000 |
|  | Scrambled SBCs | Northeast | 0.6228 | 0.0672 | 0.3444 | 0.5801 | 0.6271 | 0.6697 | 0.7773 | 1000 |
|  |  | Southeast | 0.4299 | 0.0565 | 0.2292 | 0.3912 | 0.4339 | 0.4693 | 0.5699 | 1000 |
|  |  | Central | 0.2640 | 0.0515 | 0.0785 | 0.2297 | 0.2670 | 0.3004 | 0.4090 | 997 |
|  |  | West | 0.2239 | 0.0571 | 0.0238 | 0.1862 | 0.2276 | 0.2606 | 0.4106 | 857 |
| **MLJG** | Scrambled OTUs | Northeast | 0.3665 | 0.0680 | 0.1436 | 0.3237 | 0.3686 | 0.4135 | 0.5547 | 972 |
|  |  | Southeast | 0.3604 | 0.0579 | 0.1883 | 0.3193 | 0.3615 | 0.3996 | 0.5325 | 1000 |
|  |  | Central | 0.3279 | 0.0451 | 0.1565 | 0.2972 | 0.3291 | 0.3580 | 0.4407 | 1000 |
|  |  | West | 0.3464 | 0.0619 | 0.1442 | 0.3050 | 0.3511 | 0.3896 | 0.5044 | 997 |
|  | Scrambled SBCs | Northeast | 0.4397 | 0.0960 | 0.1104 | 0.3773 | 0.4446 | 0.5026 | 0.7856 | 988 |
|  |  | Southeast | 0.3774 | 0.0611 | 0.1731 | 0.3382 | 0.3788 | 0.4222 | 0.5508 | 1000 |
|  |  | Central | 0.2554 | 0.0665 | 0.0156 | 0.2148 | 0.2606 | 0.3046 | 0.4317 | 960 |
|  |  | West | 0.2125 | 0.0674 | -0.0191 | 0.1664 | 0.2129 | 0.2580 | 0.4333 | 818 |

Abbreviations: ESV, exact sequence variant; OTU, operational taxonomic unit; SBC, species bound cluster.

**Table S11. Comparison of SBCs with F230R and MLJG amplicons.**

|  | **Spearman's correlation** | | | **F230R** | | | **MLJG** | | |
| --- | --- | --- | --- | --- | --- | --- | --- | --- | --- |
| **Species** | **ρ** | **p-value** | **p-value adjusted** | **pseudo-F** | **p-value** | **p-value adjusted** | **pseudo-F** | **p-value** | **p-value adjusted** |
| **Rhyacophila brunnea** | 1 | 0.0062 | 0.014725 | 0.553571 | 0.6435 | 0.719206 | 0.553571 | 0.6349 | 0.670172 |
| **Yoraperla brevis** | 0.914446 | 0.0015 | 0.004385 | 108.2143 | 0.0021 | 0.01596 | 5.42E+17 | 1.00E-04 | 0.000475 |
| **Ameletus validus** | 0.839336 | 1.00E-04 | 0.000543 | 5.757576 | 0.0204 | 0.0456 | 4.928064 | 0.0182 | 0.043225 |
| **Optioservus sp. BOLD:AAN4596** | 0.798523 | 1.00E-04 | 0.000543 | 2.541648 | 0.0337 | 0.071144 | 1.977925 | 0.1138 | 0.196564 |
| **Setvena bradleyi** | 0.660771 | 0.0029 | 0.007871 | 0.130252 | 1 | 1 | 0.70915 | 0.6906 | 0.709265 |
| **Drunella doddsii** | 0.619397 | 1.00E-04 | 0.000543 | 27.25221 | 1.00E-04 | 0.001267 | 21.68685 | 1.00E-04 | 0.000475 |
| **Rhyacophila hyalinata** | 0.612162 | 1.00E-04 | 0.000543 | 2.540231 | 0.0983 | 0.155642 | 1.747031 | 0.214 | 0.301185 |
| **Isogenoides colubrinus** | 0.572914 | 1.00E-04 | 0.000543 | 5.430256 | 0.0031 | 0.019633 | 2.070774 | 0.0296 | 0.0592 |
| **Brillia sp. BOLD:AAN2569** | 0.543337 | 2.00E-04 | 0.00095 | 3.891948 | 0.0067 | 0.036371 | 2.294779 | 0.0977 | 0.17679 |
| **Zapada columbiana** | 0.534477 | 1.00E-04 | 0.000543 | 2.726954 | 0.0161 | 0.0456 | 8.276657 | 1.00E-04 | 0.000475 |
| **Lepidostoma pluviale** | 0.442501 | 0.03 | 0.048925 | 5.460237 | 0.0429 | 0.0858 | 1.481161 | 0.2446 | 0.31209 |
| **Capnia sp. BOLD:ACK8742** | 0.367125 | 0.0309 | 0.048925 | -2.03432 | 1 | 1 | 1.542849 | 0.2851 | 0.338556 |
| **Lepidostoma cascadense** | 0.357099 | 0.0066 | 0.014753 | 5.334137 | 0.0184 | 0.0456 | 30.63045 | 6.00E-04 | 0.002073 |
| **Orthocladius sp. BOLD:AAN4801** | 0.319427 | 0.0187 | 0.034562 | 1.043184 | 0.4127 | 0.47523 | 1.67549 | 0.1678 | 0.2565 |
| **Diphetor hageni** | 0.315302 | 0.0191 | 0.034562 | 3.901639 | 0.0511 | 0.09709 | 7.721642 | 0.0054 | 0.014657 |
| **Prostoia besametsa** | 0.293036 | 1.00E-04 | 0.000543 | 2.101944 | 0.1475 | 0.215577 | 4.770206 | 6.00E-04 | 0.002073 |
| **Micropsectra sp. BOLD:AAM6225** | 0.282859 | 5.00E-04 | 0.0019 | 12.54156 | 1.00E-04 | 0.001267 | 2.590671 | 0.026 | 0.054889 |
| **Ameletus celer** | 0.27054 | 4.00E-04 | 0.001689 | 2.510602 | 0.0113 | 0.04294 | 1.857494 | 0.2504 | 0.31209 |
| **Eucapnopsis brevicauda** | 0.26019 | 0.0011 | 0.003483 | 2.019032 | 0.0957 | 0.155642 | 1.506647 | 0.2546 | 0.31209 |
| **Orthocladius sp. BOLD:AAN2571** | 0.258309 | 9.00E-04 | 0.003109 | 1.813574 | 0.185 | 0.242414 | 5.805719 | 1.00E-04 | 0.000475 |
| **Drunella grandis** | 0.224654 | 0.1054 | 0.154046 | 0.621345 | 1 | 1 | 0.88087 | 0.474 | 0.545818 |
| **Zapada haysi** | 0.195006 | 0.0048 | 0.01216 | 3.816571 | 0.0097 | 0.04294 | 5.039969 | 1.00E-04 | 0.000475 |
| **Pericoma sp. BOLD:AAU4662** | 0.161929 | 0.0075 | 0.015833 | 2.741529 | 0.0162 | 0.0456 | 5.125709 | 0.001 | 0.002923 |
| **Plumiperla diversa** | 0.139222 | 0.0175 | 0.034562 | 1.638021 | 0.165 | 0.232222 | 3.307263 | 0.0062 | 0.015707 |
| **Baetis tricaudatus** | 0.132716 | 0.0656 | 0.099712 | 11.33676 | 1.00E-04 | 0.001267 | 16.85561 | 1.00E-04 | 0.000475 |
| **Kogotus modestus** | 0.124922 | 0.1161 | 0.1634 | 6.888753 | 4.00E-04 | 0.0038 | 1.434146 | 0.1718 | 0.2565 |
| **Rhithrogena robusta** | 0.121073 | 0.0273 | 0.047155 | 3.571651 | 0.0142 | 0.0456 | 5.130907 | 6.00E-04 | 0.002073 |
| **Epeorus deceptivus** | 0.087236 | 0.1504 | 0.197076 | 2.125698 | 0.0572 | 0.103505 | 3.299523 | 7.00E-04 | 0.002217 |
| **Hydrobaenus sp. BOLD:ACG3277** | 0.086665 | 0.1498 | 0.197076 | 1.163111 | 0.3864 | 0.45885 | 1.963903 | 0.1245 | 0.205696 |
| **Neoleptophlebia heteronea** | 0.080867 | 0.2295 | 0.2907 | 4.19576 | 0.0607 | 0.104845 | 2.402863 | 0.0761 | 0.14459 |
| **Tipula sp. BOLD:AAG4531** | 0.018823 | 0.3195 | 0.391645 | 2.926851 | 0.0192 | 0.0456 | 2.847231 | 0.0195 | 0.043588 |
| **Arctopsyche grandis** | -0.00489 | 0.4924 | 0.584725 | 3.400233 | 0.0188 | 0.0456 | 12.77905 | 1.00E-04 | 0.000475 |
| **Zapada cinctipes** | -0.0351 | 0.6298 | 0.725224 | 1.5 | 0.2161 | 0.273727 | 23.55585 | 1.00E-04 | 0.000475 |
| **Micrasema bactro** | -0.09833 | 1 | 1 | 9.448905 | 0.011 | 0.04294 | 0.214286 | 1 | 1 |
| **Micropsectra sp. BOLD:ABY0413** | -0.11205 | 1 | 1 | 1.297814 | 0.2454 | 0.300813 | 1.071429 | 0.5372 | 0.6004 |
| **Conchapelopia pallens** | -0.12962 | 1 | 1 | 1.909091 | 0.1833 | 0.242414 | 2.757576 | 0.1755 | 0.2565 |
| **Megarcys watertoni** | -0.15406 | 1 | 1 | 1.933884 | 0.109 | 0.16568 | 1.335805 | 0.2533 | 0.31209 |
| **Tanytarsus sp. BOLD:ACB8144** | -0.18272 | 0.8 | 0.894118 | 0.2 | 0.9 | 0.977143 | 2.459692 | 0.6 | 0.651429 |

Abbreviations: SBC, species bound cluster.
