# Supplementary material for: Biomonitoring 2.0 Refined: observing local change through metaphylogeography using a community-based eDNA metabarcoding monitoring network
Source: BMC Biol. 2025 Jul 1;23:187. doi: 10.1186/s12915-025-02284-x (PMC12220750; doi:10.1186/s12915-025-02284-x)
Supplement: Supplementary file 2 — Additional file 2: Figures S1–S8. Fig. S1 Schematic of multiple sequence alignment filtering. Fig. S2 Schematic of ESV merging by cluster and dissimilarity matrix generation. Fig. S3 Schematic of ESV grouping by cluster and mean dissimilarity matrix generation. Fig. S4 Schematic of ESV scrambling by cluster to generate scrambled clusters. Fig. S5 Comparisons of adjusted Rand index using different numbers of cluster centers to separate sites into region groups. Fig. S6 Comparisons of total within-cluster sum of squares using different numbers of cluster centers to separate sites into region groups. Fig. S7 Intraspecific genetic variation separates region groups with dissimilarity patterns that differ from community β-diversity (MLJG). Fig. S8 Multiple sequence alignment of Yoraperla brevis ESVs and barcodes. [file 12915_2025_2284_MOESM2_ESM.docx]

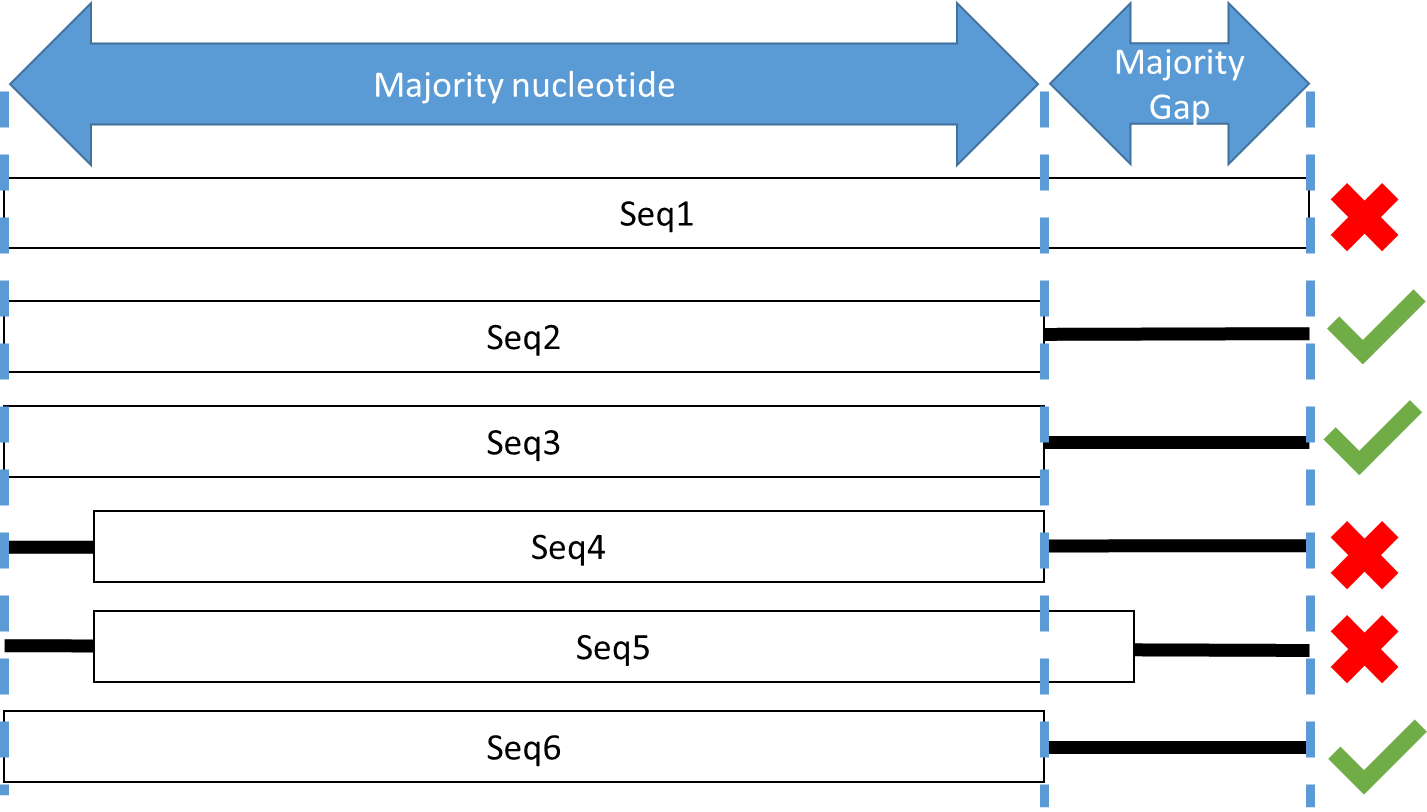


**Figure S1. Schematic of multiple sequence alignment filtering.** White rectangles represent nucleotides and black lines represent gaps. Green checks represent sequences that pass filtering and red Xs represent sequences that are filtered.


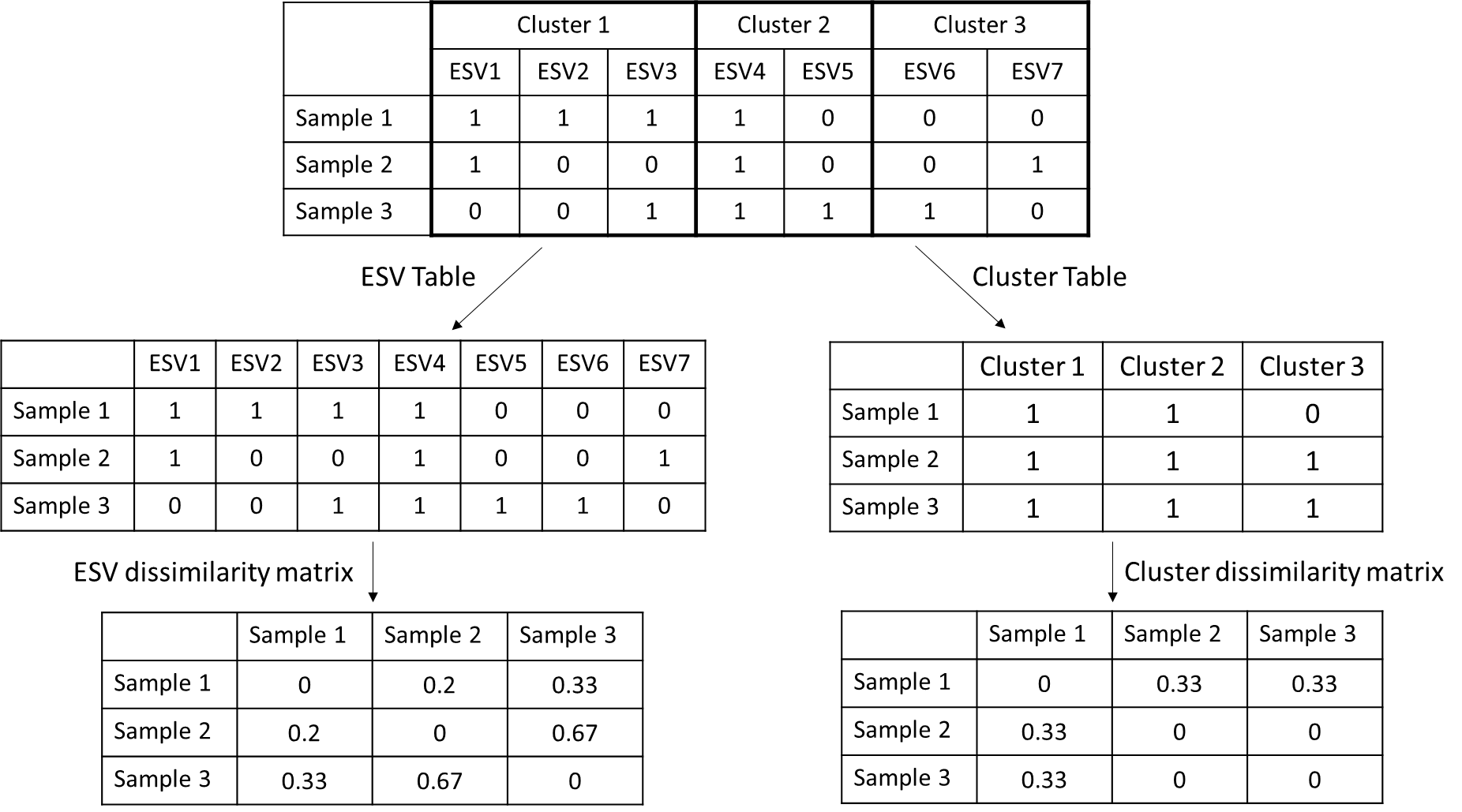


**Figure S2. Schematic of ESV merging by cluster and dissimilarity matrix generation.** Abbreviations: ESV, exact sequence variant.

**
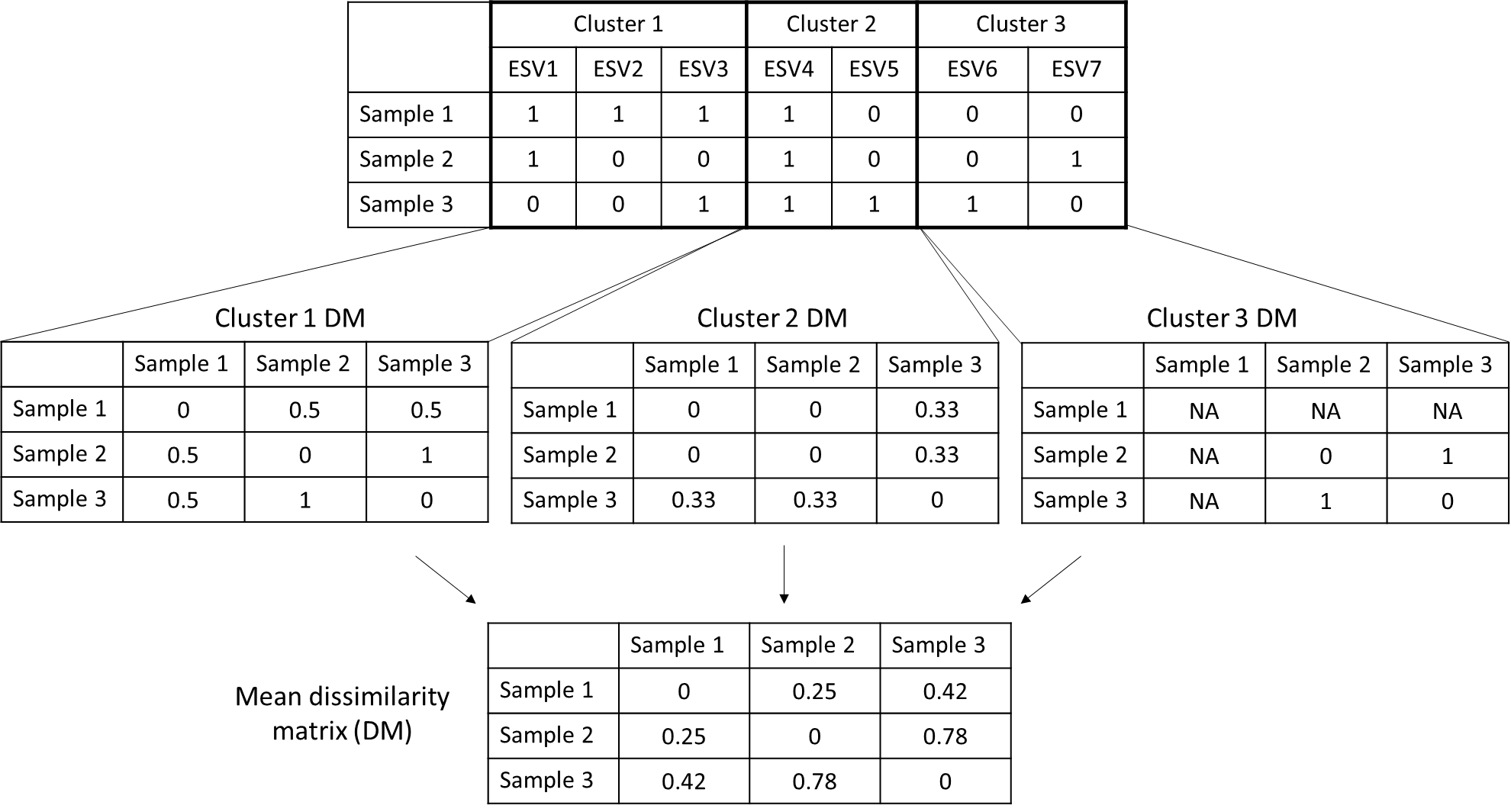
**

**Figure S3. Schematic of ESV grouping by cluster and mean dissimilarity matrix generation.** Abbreviations: ESV, exact sequence variant; DM, dissimilarity matrix.

**
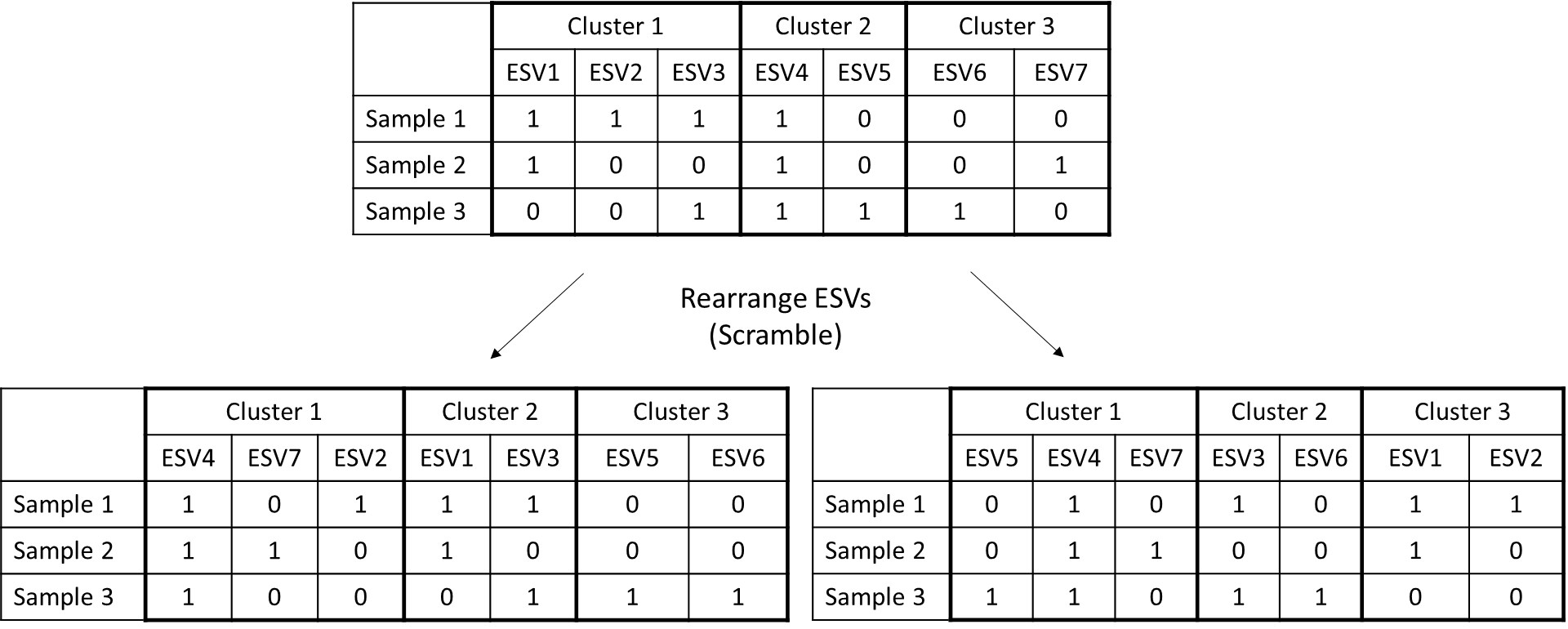
**

**Figure S4. Schematic of ESV scrambling by cluster to generate scrambled clusters.** Abbreviations: ESV, exact sequence variant.


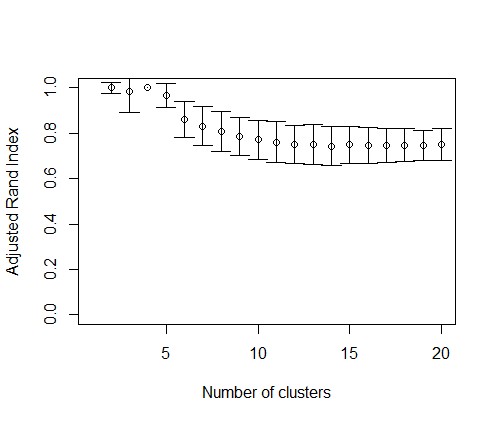


**Figure S5. Comparisons of adjusted Rand index using different numbers of cluster centers to separate sites into region groups.**


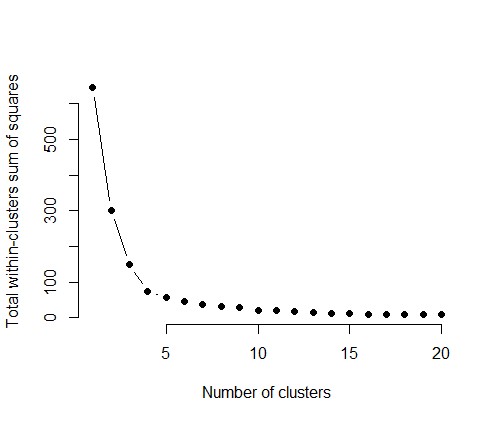


**Figure S6. Comparisons of total within-clusters sum of squares using different numbers of cluster centers to separate sites into region groups.** Figure was generated based on code from https://uc-r.github.io/kmeans_clustering.


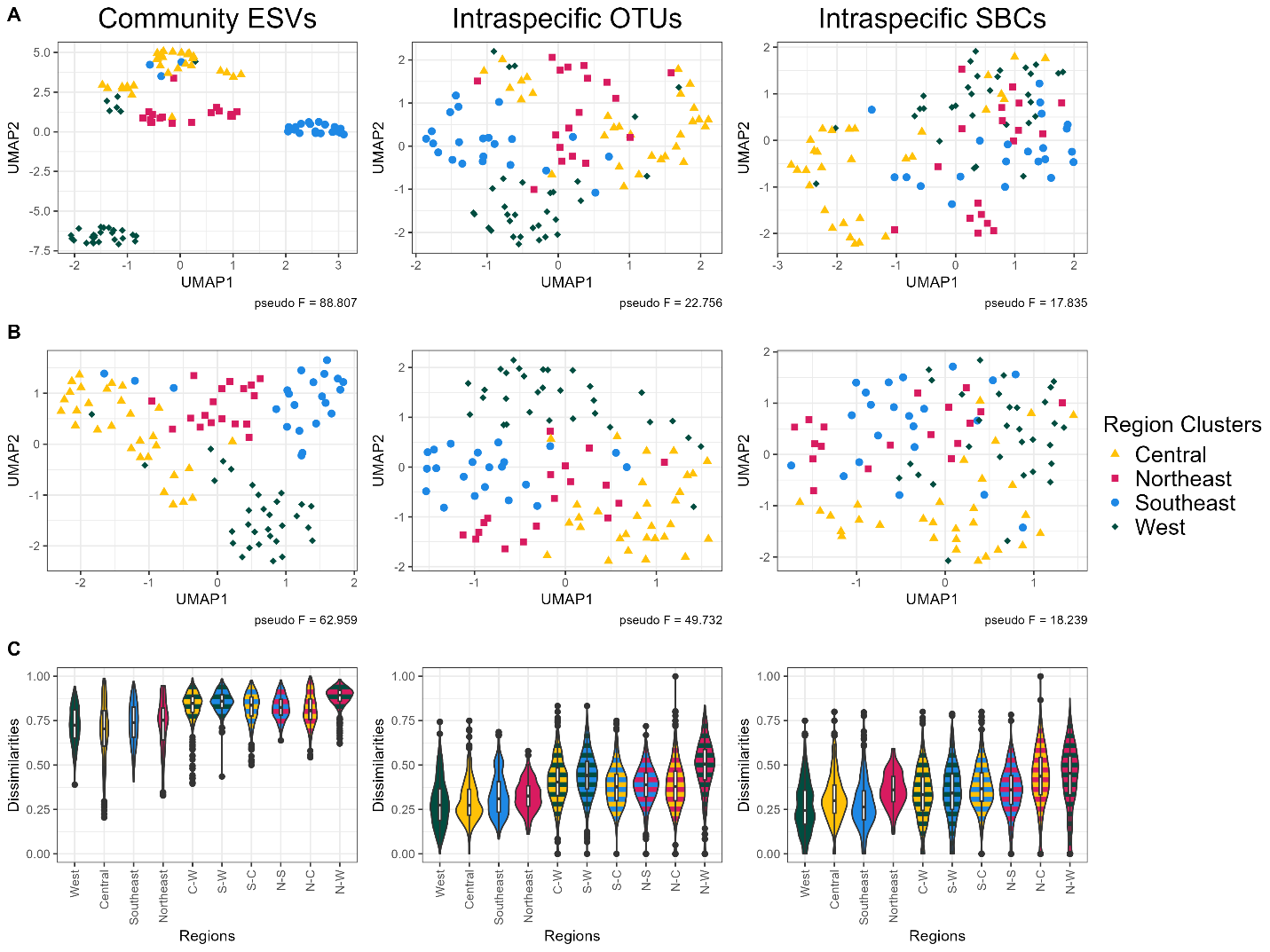


**Figure S7. Intraspecific genetic variation separates region groups with dissimilarity patterns that differ from community β-diversity (MLJG)**. Uniform manifold approximation and projection (UMAP) using 15 neighbours (A), using max neighbours (number of samples – 1) (B), and violin plots of dissimilarities (C) were generated to compare the community ESVs to intraspecific OTUs and SBCs from amplicon MLJG. Pseudo *F*-statistics were calculated using PERMANOVA for the separation of region clusters based on UMAP ordinations. All PERMANOVA tests were significant (*p*-value < 0.05). Abbreviations: C-W, Central-West; ESV, exact sequence variant; N-C, Northeast-Central; N-S, Northeast-Southeast; N-W, Northeast-West; OTU, operational taxonomic unit; SBC, species bound cluster; S-C, Southeast-Central; S-W, Southeast-West.


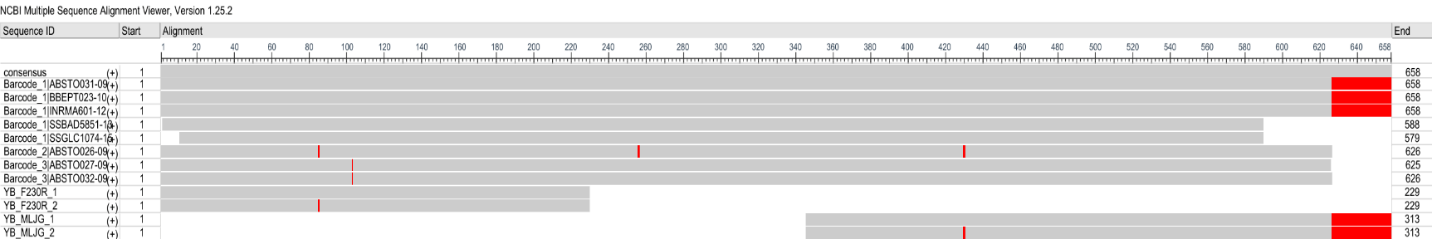


**Figure S8. Multiple sequence alignment of *Yoraperla brevis* ESVs and barcodes.** Red bars indicate nucleotides that differ from the consensus. Multiple sequence alignment was created using MAFFT and visualized using NCBI Multiple Sequence Alignment Viewer (1.25.2). Jeffery M. Webb (ABSTO026-09) - Center for Biodiversity Genomics; Jeffery M. Webb (ABSTO027-09) - Center for Biodiversity Genomics; Jeffery M. Webb (ABSTO031-09) - Center for Biodiversity Genomics; Jeffery M. Webb (ABSTO032-09) - Center for Biodiversity Genomics; Jeffery M. Webb (BBEPT023-10) - Center for Biodiversity Genomics; Jayme E. Sones (INRMA601-12) - Royal British Columbia Museum; (SSBAD5851-13) - Center for Biodiversity Genomics; Kate Perez (SSGLC1074-15) - Center for Biodiversity Genomics. (Accessed on 2024-12-12) via boldsystems.org.
